# PIKFYVE inhibitors trigger interleukin‐24‐dependent cell death of autophagy‐dependent melanoma

**DOI:** 10.1002/1878-0261.13607

**Published:** 2024-02-27

**Authors:** Ajit Roy, Arup R. Chakraborty, Melvin L. DePamphilis

**Affiliations:** ^1^ National Institute of Child Health & Human Development National Institutes of Health Bethesda MD USA

**Keywords:** autophagy, endoplasmic‐reticulum‐stress, interleukin‐24, PIKFYVE, PIP4K2C, WX8

## Abstract

Inhibitors specifically targeting the 1‐phosphatidylinositol 3‐phosphate 5‐kinase (PIKFYVE) disrupt lysosome homeostasis, thereby selectively terminating autophagy‐dependent human cancer cells *in vivo* as well as *in vitro* without harming the viability of nonmalignant cells. To elucidate the mechanism by which PIKFYVE inhibition induces cell death, autophagy‐dependent melanoma cells were compared with normal foreskin fibroblasts. RNA sequence profiling suggested that PIKFYVE inhibitors upregulated an endoplasmic reticulum (ER) stress response involving interleukin‐24 (IL24; also known as MDA7) selectively in melanoma cells. Subsequent biochemical and genetic analyses confirmed these results and extended them to tumor xenografts in which tumor formation and expansion were inhibited. *IL24* expression was upregulated by the DDIT3/CHOP/CEBPz transcription factor, a component of the PERK‐dependent ER‐stress response. Ectopic expression of IL24‐induced cell death in melanoma cells, but not in foreskin fibroblasts, whereas ablation of the *IL24* gene in melanoma cells prevented death. *IL24* upregulation was triggered specifically by PIKFYVE inhibition. Thus, unlike thapsigargin and tunicamycin, which induce ER‐stress indiscriminately, PIKFYVE inhibitors selectively terminated PIKFYVE‐sensitive melanoma by inducing IL24‐dependent ER‐stress. Moreover, induction of cell death by a PIKFYVE inhibitor together with ectopic expression of IL24 protein was cumulative, thereby confirming the therapeutic potential of PIKFYVE inhibitors in the treatment of melanoma.

## Graphical abstract

PIKFYVE inhibitors selectively kill melanoma cells by disrupting lysosome homeostasis, which triggers an ER‐stress response that upregulates expression of glycosylated interleukin‐24 (IL24). IL24 amplifies the ER‐stress response to induce cell death. Thapsigargin and tunicamycin also trigger ER‐stress, but they are toxic to normal cells as well as cancer cells.

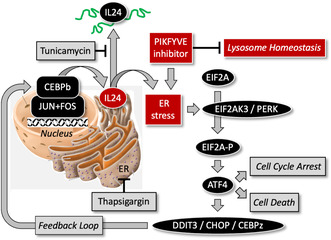

**Abbreviations**
Adr: Adriamycin
Apl: apilimod
BafA1: bafilomycin‐A1
CQ: chloroquine
DMSO: dimethyl sulfoxide
ELISA: enzyme‐linked immunosorbent assay
ER: endoplasmic reticulum
Etop: etoposide
FACS: fluoresce activated cell sorting
IL24: interleukin‐24
ns: not significant
nt‐siRNA: nontarget siRNA
PCR: polymerase chain reaction
pfu: plaque forming units
PI(3,5)P_2_
: phosphatidyl inositol 3,5‐bisphosphate
PI(4)P: phosphatidyl inositol 4‐phosphate
PI(4,5)P_2_
: phosphatidyl inositol 4,5‐bisphosphate
PI(5)P: phosphatidyl inositol 5‐phosphate
PIKFYVE: 1‐phosphatidylinositol 3‐phosphate 5‐kinase activity
PIP4K2C: 1‐phosphatidylinositol 5‐phosphate 4‐kinase activity
PIP5K1C: 1‐phosphatidylinositol 4‐phosphate 5‐kinase activity
RNA‐seq: RNA sequence profiling
RT‐PCR: real‐time polymerase chain reaction
SD: standard deviation
SEM: standard error of the mean
STR: short tandem repeat
THAP: thapsigargin
TUN: tunicamycin
V: vehicle (DMSO)
Vac: Vacuolin‐1
WT: wild‐type
YM: YM201636

## 1 Introduction

The PIKFYVE phosphoinositide kinase phosphorylates phosphatidylinositol 3‐monophosphate [PI(3)P] into phosphatidylinositol‐3,5‐bisphosphate [PI(3,5)P_2_], a phosphoinositide involved in lysosome fission, trafficking of molecules into lysosomes, endolysosomal trafficking, and fusion between autophagosomes and lysosomes [1]. All of these events are involved macro‐autophagy [2]. Thus, inhibitors of PIKFYVE inhibit autophagy and prevent the growth of cancer cells both *in vitro* and *in vivo*. Examples include lymphoma [3, 4], liver [5], multiple myeloma [6], colorectal [7], prostate [8], embryonal carcinoma [9], gastric [10], and melanoma (this report). Thus, PIKFYVE inhibitors can selectively terminate autophagy‐dependent cancer cells and pluripotent cancer stem cells under conditions where nonmalignant cells remain viable [4, 7, 9, 11, 12, 13, 14].

Cells that are sensitive to PIKFYVE inhibitors are deficient in PIP5K1C, a phosphoinositide kinase that converts phosphatidyl inositol 4‐phosphate [PI(4)P] into PI(4,5)P_2_, a phosphoinositide essential for macro‐autophagy [13]. PI(4,5)P_2_ is produced via two independent pathways [2, 15, 16]. One requires PIP5K1C to convert PI(4)P into PI(4,5)P_2_. The other requires PIKFYVE to convert PI(3)P into PI(3,5)P_2_ which is then converted into PI(5)P by a 3‐phosphatase. PI(5)P is then converted into PI(4,5)P_2_ by the PIP4K2C phosphoinositide kinase [13].

WX8 is an extensively characterized small molecule inhibitor of PIKFYVE [7, 9, 13, 14, 17]. At low concentrations, WX8 specifically inhibits PIKFYVE *in situ*, whereas at higher concentrations WX8 inhibits both PIKFYVE and PIP4K2C, thereby dramatically suppressing PI(4,5)P_2_ synthesis [13]. Consequently, selective inhibition of PIKFYVE alone inhibits cell proliferation, whereas inhibition of both PIKFYVE and PIP4K2C, either with higher concentrations of WX8 or with low concentrations of WX8 together with siRNA against PIP4K2C, induces cell death [13]. However, events that trigger cell death vary with cancer cells derived from different tissues [4, 7, 13].

Skin cancer is the most common type of cancer, and melanoma is the deadliest form of skin cancer [18]. However, recent research as well as clinical trials suggest that inhibition of autophagy has therapeutic potential against melanoma [19, 20]. Therefore, a panel of previously characterized autophagy‐dependent cell lines [13] was used to identify the mechanism whereby PIKFYVE inhibitors selectively kill cancer cells. Among them, autophagy‐dependent melanoma A375 cells and normal HFF1 foreskin fibroblasts served as paradigms for PIKFYVE‐sensitive and resistant cells, respectively [14, 21, 22]. Therefore, to identify changes in gene expression linked to cell death, A375 and HFF1 cells were treated with WX8 and analyzed by RNA‐sequence profiling (RNA‐seq).

The results suggested that PIKFYVE inhibitors triggered an endoplasmic reticulum (ER) stress response involving interleukin‐24 (IL24/MDA7) selectively in PIKFYVE‐sensitive cells. IL24 is a N‐linked glycosylated multifunctional cytokine present in many types of cells [23]. Subsequent biochemical and genetic analyses revealed that PIKFYVE inhibitors induced a PERK‐dependent ER‐stress response that upregulated IL24 expression, which resulted in the termination of autophagy‐dependent melanoma cells *in vivo* as well as *in vitro*. These results were consistent with previous studies showing that ectopic expression of IL24 can induce growth arrest and DNA damage‐inducible genes characteristic of PERK‐dependent ER‐stress with concomitant induction of apoptosis [24, 25, 26, 27, 28]. The combined effects of PIKFYVE inhibitors and ectopic expression of IL24 protein were additive, thereby confirming the therapeutic potential of elevating IL24 levels in the treatment of melanoma. Inhibition of PIKFYVE activity induced IL24 expression with concomitant amplification of the PERK‐dependent ER‐stress response, which resulted in the termination of autophagy‐dependent melanoma cells *in vivo* as well as *in vitro*.

## 2 Materials and methods

### 2.1 Cells and culture

Normal [HFF1 (CVCL_3285), Hs27 (CVCL_0335), 293T (CVCL_0063)] and malignant [Melanoma A375 (CVCL_0132), U20S (CVCL_0042), HCT116 (CVCL_0291), MeWo (CVCL_0445)] human cell lines were purchased from the American Type Culture Collection and cultured as described [13]. Melanoma cell lines [M321 (CVCL_D768), M257 (CVCL_D757) and M238 (CVCL_D751)] were obtained from Dr. Antoni Ribas (Department of Medicine, University of California, Los Angeles, CA) and cultured and authenticated as described [29]. The ATCC has authenticated its cell lines during the past 3 years by comparing the STR profile of sample cell lines with their Human Cell STR Database. All cell lines were tested for mycoplasma periodically using ATCC universal Mycoplasma Detection Kit (30‐1012 K). All experiments were performed with mycoplasma free cells.

### 2.2 Standard assays

Immunoblotting to detect and quantify protein levels, FACS analysis to quantify the fraction of cells with < 2N DNA content, and trypan blue assays to quantify dead cells were done as previously described [9, 13, 14].

### 2.3 Antibodies, inhibitors, and reagents

Antibodies were from Cell Signaling Technology (Danvers, MA, USA) [SQSTM1/p62 (7114), LC3 I/II (12741), CASP3 (9662S), cleaved‐CASP3 (9664), PARP (9542S), ACTB (SAB4200248), ATF4 (11815S), DDIT3 (2895 s), XBP1s (12782), ATF6 (65880), LMNB1 (12586S), Phospho‐Histone H2A.X (2577), cleaved‐PARP (5625S)], from Abcam (Boston, Waltham, MA, USA) [Vinculin (ab91459) and EIF2A (ab169528)], and (IL24, K101) from GenHunter (Nashville, TN, USA).

WX8; 1H‐indole‐3‐carbaldehyde [4 anilino 6 (4 morpholinyl) 1,3,5 triazin 2 yl]hydrazone was purchased from SPECS, Point Judith, RI, USA (PubChem number MLS000543798; pubchem.ncbi.nlm.nih.gov). WX8 is also available from Millipore Sigma (Burlington, VT, USA). Thapsigargin (HY 13433), tunicamycin (HY A0098), apilimod (HY 14644), Vacuolin‐1 (HY 118630), and YM 201636 (HY 13228) were from Medchem Express (Monmouth Junction, NJ, USA). CeapinA7 (SML2330), APY29 (1216665‐49‐4), ISRIB (1597403‐47‐8), Adriamycin/Doxorubicin hydrochloride (25316‐40‐9), Chloroquine (50‐63‐5), MKC 3946 (532758), and GSK2656157 (504651) and Trypan Blue (T6146) were from Sigma‐Aldrich (St. Louis, MO, USA). PNGase F (P0704) was from New England Biolabs (Ipswich, MA, USA). NCT504 was from MedChemExpress (HY‐136311).

### 2.4 RNA sequence profiling

RNA isolation and RNA sequence profiling of A375 and HFF1 cells were carried out as previously described [13]. Cells were seeded at 2000 cells·cm^−2^ and cultured for 16–18 h. Cells were then cultured 24 h in the presence of 0.05 μm WX8, 1 μm WX8, or an equivalent amount of dimethyl sulfoxide (DMSO), the vehicle used to dissolve WX8. A375 cells were analyzed in quadruplicates. HFF1 cells were analyzed in triplicate. RNA‐seq data on the expression of genes associated with the IL24 ER‐stress response are presented here. RNA‐seq data on the expression of phosphoinositide kinases associated with lysosome homeostasis and autophagy were published previously [13].

### 2.5 Real‐Time PCR (RT‐PCR)

Total RNA was extracted from cells and tumors using RNeasy Mini Kit (Qiagen, Germantown, MD, USA, #74104). cDNA synthesis was performed on 1 μg of total cellular RNA using SuperScript™ III First‐Strand Synthesis SuperMix cDNA synthesis kit (Thermo Fisher Scientific, Waltham, MA, USA, #18080400). IL24, SQSTM1 and GAPDH RNAs were assayed using Amplitaq Gold Mastermix (Thermo Fisher Scientific, #4398881). Relative quantification of genes was performed using the ΔΔ*C*_t_ method [30].

### 2.6 siRNA transfection

ON‐TARGET smart pool siRNAs were purchased from Dharmacon and reverse transfected into cells using Lipofectamine RNAiMax (Thermo Fisher Scientific, 13778075) according to the manufacturer's instructions. Cells were seeded at 2631 cells·cm^−2^ in 24 well plates during transfection. At 24 h post‐transfection, cells were treated either with 1 μm WX8 or an equivalent volume of the DMSO vehicle. Cells were cultured for 72 h before collecting the culture medium and removing the attached cells by trypsinization. The total cell population was lysed, and then analyzed for the indicated proteins by immunoblotting whole cell extracts as described [13]. DDIT3 siRNAs were GGUAUGAGGACCUGCAAGA, CACCAAGCAUGAACAAUUG, GGAAACAGAGUGGUCAUUC, and CAGCUGAGUCAUUGCCUUU. IL24 siRNAs were UCAAACAGUUGGACGUAGA, GCACACAGGCGGUUUCUGC, ACUAUAACCUUGUUCCAAA, and GAAGGCAGCAGAAUAUUGU. Nontarget control ntsiRNAs were UGGUUUACAUGUCGACUAA, UGGUUUACAUGUUGUGUGA, UGGUUUACAUUUUUCUGA, and UGGUUUACAUGUUUUCCUA.

### 2.7 Xenografts

Inbred female BALB/c nu/nu (CAnN.Cg‐*Foxn1nu*/Crl) mice (Charles River Laboratories, Wilmington, MA, USA) and outbred female BALB/c nu/nu (J:NU) nude mice (Jackson Laboratory, Bar Harbor, ME, USA, 007850) were used for xenografts [9, 31]. Female CD1 IGS [Crl:CD1(ICR)] mice 6–8 weeks old (Charles River) were used for toxicity tests. Housing and handling conditions of animals are described in Animal Study Proposal 21‐056 approved by the NICHD/NIH.

All procedures were performed under general anesthesia with isoflurane and conducted under studies approved by the NICHD Animal Care and Use Committee.

Stock solutions of 206.8 mm WX8 were prepared by dissolving the maximum amount of WX8 in DMSO (30 mg WX8/350 μL DMSO) without producing a visible precipitate. DMSO is toxic. Therefore, to minimize the amount of DMSO in intraperitoneal injections, 10 μL WX8 stock solution was dissolved in a total volume of ≤ 150 μL sunflower seed oil (Millipore Sigma, S5007). Prior to injection, the yellow color should be thoroughly mixed with the oil.

Melanoma A375 cells were cultured until 80% confluent. Cells were then trypsinized and stained with trypan blue to quantify the fraction of live (unstained) cells [32]. Live cells (10^6^/100 μL) in ice cold DMEM culture medium without FBS were then mixed with an equal volume of Matrigel and then subcutaneously inoculated into both the flanks of nude mice using Coviden 1 mL syringe with 22 gauge 1″ needle [9, 31]. Palpable tumors (40–50 mm^3^) appeared within 6–7 days postinoculation. Mice then received daily intraperitoneal injections of either vehicle, 20 mg WX8, or 40 mg WX8 per kg mouse using a 28G insulin syringe (EXELlINT, 26027) for 14 days before being euthanized and tumors excised. Tumor sizes abided by the US animal welfare guidelines and never interfered with mouse locomotion, eating, or drinking.

Tumor tissues were fixed overnight either at 4 °C in Bouin's fixative, or at room temperature in fresh 4% paraformaldehyde in phosphate‐buffered saline, embedded in paraffin, and sectioned into 5 micron slices. Slides were then removed from paraffin using xylene, washed with alcohol, and hydrated by thoroughly rinsing with water. Sections were then stained first with Harris hematoxylin to stain nuclei, rinsed with alcohol, and then with eosin to stain cytoplasm. Images were obtained using a Nanozoomer (Hamamatsu Photonics, Wilmington, MA, USA). Tumor lysates were prepared after mincing and grinding tumor slices frozen in liquid nitrogen with a mortar and pestle followed by lysis in SDS‐lysis buffer without dithiothreitol. Protein was quantified using Pierce BCA Protein Assay Kit (Thermo Fisher Scientific, 23225).

### 2.8 ELISA assays

Cells were seeded at 50 000 cells per well in a 6‐well plate, and 16–18 h later treated with either vehicle or WX8 and then cultured for 72 h. The culture medium was then centrifuged at 18 000 ***g*** for 15 min at 4 °C to remove any detached cells, diluted and IL24 quantified using the IL24 ELISA Kit (OriGene, Rockville, MD, USA, EA100090) and manufacturer's instructions.

### 2.9 CRISPR‐Cas9‐mediated *IL24* gene ablation

Exon‐2 in the *IL24* gene was ablated in melanoma A375 cells using a Gene Knockout Kit v2 (Synthego, Redwood City, CA, USA) with multi‐guide sgRNA's (gRNA1‐GAGGCUGUCGCCAGCAA, gRNA2‐CUCUGGAGCCAGGUAUC, gRNA3‐ACCCCCUUCACUUGGCA). To optimize the knockout efficiency, cells were seeded at different densities in a 24‐well plate. A mixture of three different modified sgRNA's were mixed with purified CAS9 and then transfected into cells using Lipofectamine™ CRISPRMAX™ Cas9 Transfection Reagent (Thermo Fisher Scientific, CMAX00001). After 72 h, cells with different seeding densities were checked for CRISPR edits using exon‐specific PCR and analysis using the ICE CRISPR tool. Cells with the most CRISPR edits were then cloned by serially diluting them into 96‐well plates and then cultured until colonies formed. Individual colonies were checked for *IL24* gene expression by immunoblotting to detect IL24 protein. Clones with no *IL24* expression were then checked for ablation of the *IL24* gene using exon‐specific PCR.

### 2.10 Ectopic expression of AdV‐*IL24*

The replication defective adenovirus expression vector Ad.*mda*‐*7* (here termed AdV‐*IL24*) was propagated in 293T cells as described [33, 34, 35]. AdV‐*IL24* expressed the human IL24 protein whereas AdV‐null was empty. Cells (0.3 × 10^5^) were seeded into 24‐well plates and cultured overnight. AdV‐*IL24* and AdV‐null stocks were thawed and kept on ice. The desired number of plaque forming units (pfu) were added to serum‐free, antibiotic‐free, medium in a polystyrene tube and mixed by either gentle vortex or pipetting. The culture medium was removed by aspiration, and the adenovirus (~ 200 μL per well) added to the cells. The plate was gently rocked back and forth several times and then cultured in 5% CO_2_ for 2 h at 37 °C. Cells were cultured at 37 °C in 5% CO_2_. To insure even distribution of the virus, the plate was rocked gently back and forth once every 15 min for a period of 2 h. Without removing the infection mixture, 1.8 mL of standard culture medium containing 10% fetal calf serum was added and culturing continued for 72 h.

### 2.11 Statistical calculations

Either the standard deviation (SD) or the standard error of the mean (SEM) is indicated for each graph. *P*‐values in RNA‐seq were determined by Wald Chi‐square test using the DEseq2 platform. *P*‐values of two data sets from the other experiments were calculated by Student's paired two‐tailed *t*‐test using graphpad prism (GraphPad, Boston, MA, USA).

## 3 Results

### 3.1 RNA sequence profiling and validation

To validate conditions under which genes linked to induction of cell death by PIKFYVE inhibitors could be detected, RNA‐seq was first used to detect changes in the expression of genes known to be upregulated by PIKFYVE inhibition. These genes are upregulated because autophagic precursors accumulate, and cells respond by compensating for the autophagic deficiency.

Melanoma A375 cells were cultured for 24 h either with 0.05 μm WX8 to selectively inhibit PIKFYVE, or with 1 μm WX8 to inhibit both PIKFYVE and PIP4K2C, or with the vehicle to provide basal levels of gene expression. HFF1 foreskin fibroblasts were cultured under the same conditions in either 1 μm WX8 or vehicle.

*MAP1LC3B/LC3‐I*, *SQSTM1/p62*, *WIPI1/ATG18*, and *ATG* are autophagosome associated genes. LC3‐I protein and its post‐translational phosphatidylethanolamine adduct LC3‐II reside in autophagosome membranes. SQSTM1 is a ubiquitin‐binding protein that targets proteins to autophagosomes [36]. WIPI1 is an essential regulator of early autophagosome assembly [37]. ATG4A activates ATG8 proteins that regulate membrane elongation during autophagosome biogenesis [38]. WX8 significantly upregulated both RNA (Fig. S1A) and protein (Fig. S1B) levels for these genes in a concentration‐dependent manner, as previously reported [7, 14]. Lysosomal structural proteins LAMP1 and LAMP2, cathepsin proteases, and vacuolar ATPases were also upregulated. Results from RNA‐seq were confirmed by RT‐PCR (Fig. S2A). These results were consistent with previous reports that autophagy‐linked genes are upregulated whenever autophagic flux is disrupted in mammalian cells [4, 7, 14, 39]. Furthermore, autophagosomes rapidly accumulated in PIKFYVE‐sensitive A375 cells with increasing concentrations of WX8, but not in PIKFYVE‐resistant HFF1 cells (Fig. S1C,D). This was consistent with the fact that A375 cells depend on PIKFYVE to synthesize PI(4,5)P_2_ whereas HFF1 cells do not [13].

### 3.2 WX8 activated the ‘IL24 ER‐stress’ pathway selectively in PIKFYVE‐Sensitive cells

To identify genes whose expression changed significantly upon inhibition of PIKFYVE in melanoma A375 cells, but not in human foreskin fibroblasts HFF1 cells, A375 and HFF1 cells were cultured in either vehicle, or in 0.05 μm WX8 to selectively inhibit PIKFYVE, or 1 μm WX8 to inhibit both PIKFYVE and PIP4K2C. Cells were then subjected to RNA‐seq analysis, and the results analyzed by ‘Qiagen's Ingenuity Pathway Analysis’ program. Only the ‘IL24 ER‐stress’ pathway was clearly upregulated in A375 cells but not in HFF1 cells.

**Fig. 1:**
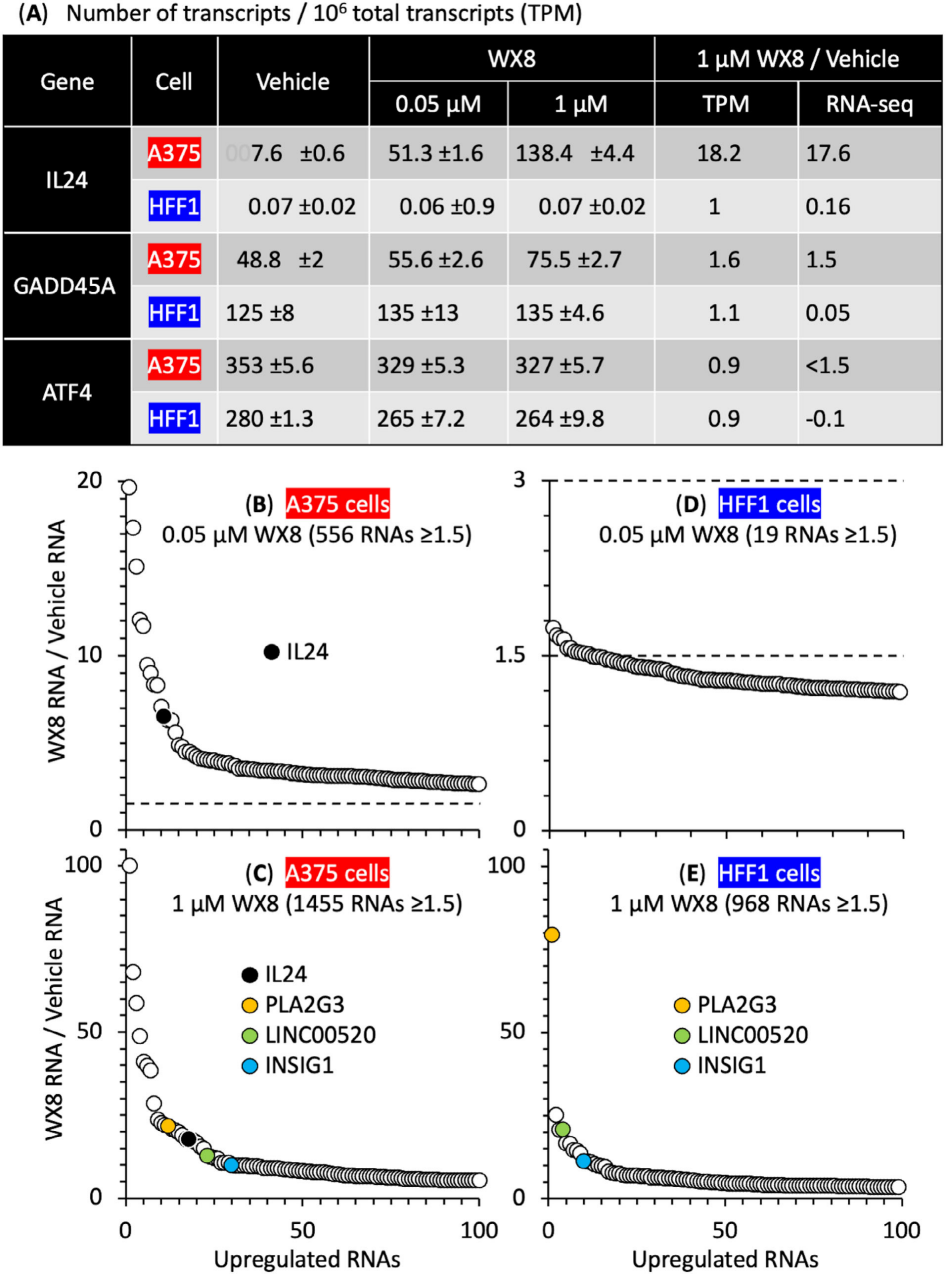
RNA‐seq analysis of genes upregulated by WX8 in melanoma A375 cells and HFF1 foreskin fibroblasts. (A) The total number of IL24 transcripts per 10^6^ total transcripts [±SD for A375 (*n* = 4) and HFF1 (*n* = 3)] in cells treated for 24 h with either vehicle, 0.05 μm or 1 μm WX8. (B, C) RNA sequence analysis identified 37 936 different RNAs in A375 cells. The 100 most highly expressed genes were plotted against their RNA abundance. The top 5% of RNAs upregulated with 0.05 μm WX8 were also present in the top 26% of RNAs upregulated with 1 μm WX8. (D, E) RNA sequence analysis identified 31 249 different RNAs in HFF1 cells. Of these, 19 were upregulated by 0.05 μm WX8, but 968 were upregulated by 1 μm WX8. IL24 was upregulated in A375 cells, but not in HFF1 cells. Only PLA2G3, LIN00520, and INSIG1 were upregulated in both cell lines. Ratios < 1.5 were considered not significant. In panels B and D, the number of genes upregulated ≥ 1.5 [fold change] greater than vehicle treated cells is marked by horizontal broken lines. Panels B and C are the average of four samples. Panels D and E are the average of three samples.

A375 cells contained 109× more IL24 transcripts than HFF1 cells (Fig. 1A). *IL24* expression was upregulated in A375 cells seven‐fold by 0.05 μm WX8 and 18‐fold by 1 μm WX8, but *IL24* expression not upregulated in HFF1 cells. RNA‐seq identified 37 936 different RNAs in A375 cells. Of these, 556 genes were upregulated in A375 cells by 0.05 μm WX8, and 1455 genes were upregulated by 1 μm WX8 (Fig. 1B,C). IL24 was among the top 2% of genes upregulated by 0.05 μm WX8 and the top 1% of genes upregulated by 1 μm WX8. In contrast, no genes were upregulated in HFF1 cells by 0.05 μm WX8, and only 187 genes were upregulated by 1 μm WX8 (Fig. 1D,E). Only *PLA2G3*, *LIN00520* and *INSIG1* were upregulated in both A375 and HFF1 cells, possibly due to induction of the SREBP transcription factors that regulate biosynthesis of lipids and cholesterol [40].

**Fig. 2:**
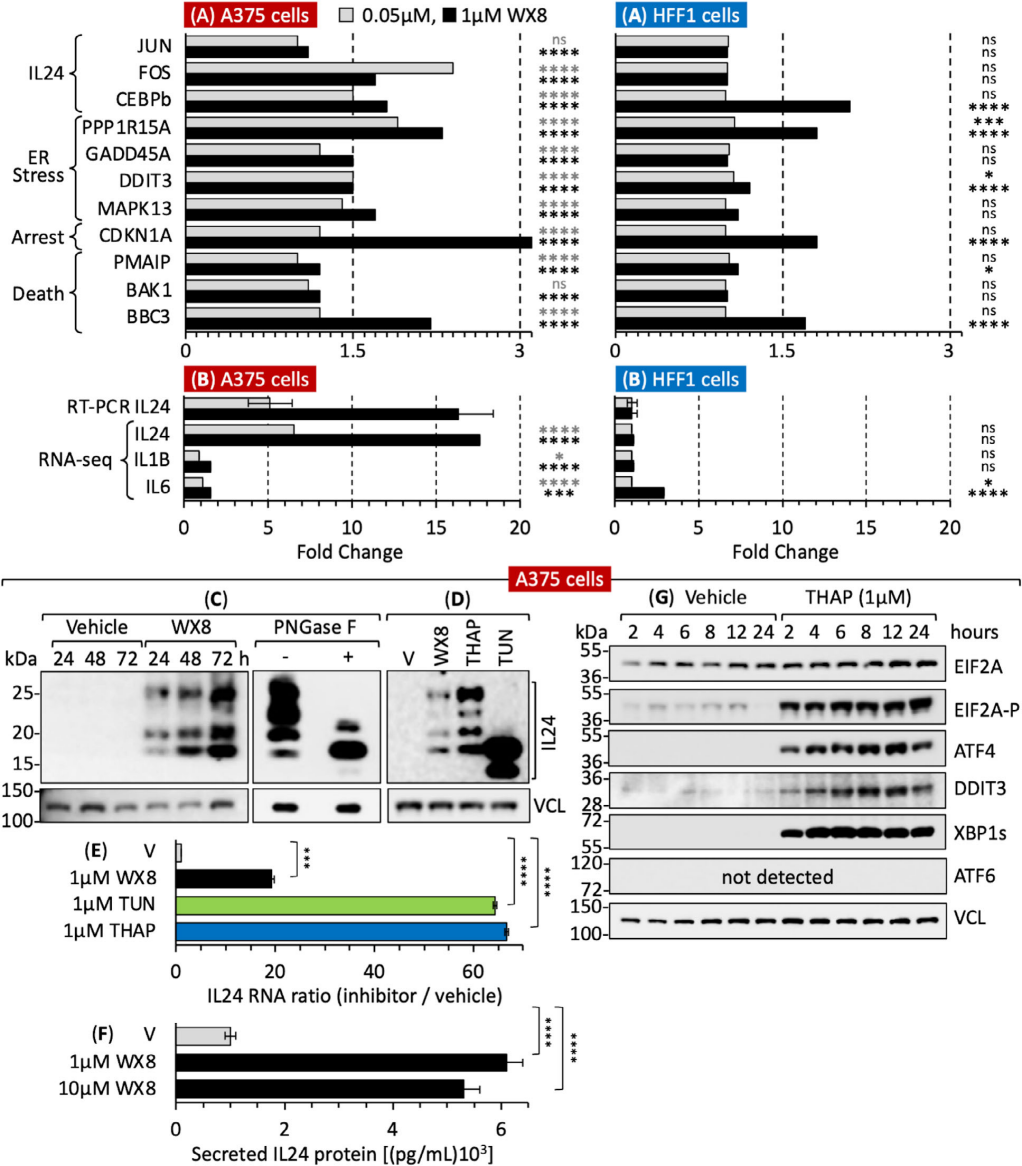
WX8 triggered the EIF2AK3/PERK‐dependent ER‐stress response. The EIF2AK3/PERK‐dependent ER‐stress response in WX8‐treated melanoma A375 cells consisted of 17 genes that were detected by upregulation of their RNA and/or protein levels (Fig. S2B). (A) RNA levels were quantified by RNA‐seq analysis in both A375 cells and HFF1 cells. Gray bars are 0.05 μm WX8. Black bars are 1 μm WX8. Genes with RNA levels ≥ 1.5 fold change above vehicle (V)‐treated cells were considered as significant upregulation. (B) Upregulation of interleukin‐24 (IL24) RNA was detected by both RT‐PCR and RNA‐seq. (C) IL24 protein was detected by immunoblotting. Extracts from WX8‐treated cells were treated with PNGase F to remove N‐linked oligosaccharides from IL24. (D) Cells treated for 24 h with vehicle (V), 1 μm WX8, 1 μm thapsigargin (THAP) or 1 μm tunicamycin (TUN) were immunoblotted for IL24 protein. (E) IL24 RNA in samples from panel D were quantified by RT‐PCR (V indicates ‘vehicle’). (F) Cells were treated with vehicle (V), 1 μm WX8 or 10 μm WX8 for 24 h. IL24 secreted into the culture medium by was quantified by ELISA assays. (G) Melanoma A375 cells were cultured with either vehicle or 1 μm thapsigargin (THAP) for the indicated times. Whole cell extracts were then immuno‐blotted for the indicated proteins, which were identified by their molecular weight and their reaction with a specific antibody. Gene symbols are from the HUGO gene nomenclature committee. V indicates ‘vehicle’. Error bars indicate ± SEM for three samples. Statistical significance was *P* < 0.05 (*), *P* < 0.001 (***), *P* < 0.0001 (****). *P* > 0.05 was ‘not significant’ (ns). *P*‐values in RNA‐seq were determined by Wald Chi‐square test using the DEseq2 platform. *P*‐values of two data sets from the other experiments were calculated by Student's paired two‐tailed *t*‐test using graphpad prism.

In addition to IL24, eight genes in the ‘PERK‐dependent ER‐stress response’ [41, 42] were also transcriptionally upregulated: *DDIT3/CHOP*, *PPP1R15A/GADD34*, *GADD45A*, *MAPK13/p38d*, *CDKN1A/p21*, *PMAIP/NOXA*, *BAK1/CDN1*, and *BBC3/PUMA* (Fig. S2B). These genes were upregulated 1.5‐ to 3‐fold above vehicle‐treated A375 cells, and the probability that the observed signal was due to chance was negligible (*P* < 0.0001, Fig. 2A). In contrast, expression of these genes in HFF1 cells treated with 0.05 μm WX8 was not significant (*P* > 0.05), and only four of these genes were upregulated significantly by 1 μm WX8 (Fig. 2A). Remarkably, RNA‐seq detected a six to eight fold upregulation of IL24 in A375 cells, but no upregulation in HFF1 cells, results that were confirmed by RT‐PCR (Fig. 2B). This effect appeared specific for IL24 because WX8 did not upregulate either IL1B or IL6. Treatment of cell extracts with the N‐linked specific glycosylase PNGase F reduced IL24 protein to its molecular weight of 18 kDa (Fig. 2C), thereby revealing that WX8 upregulated expression of IL24 protein with multiple levels of glycosylation. Upregulated IL24 protein was then secreted into the cell culture medium (Fig. 2F).

To determine whether or not upregulating IL24 expression was a consequence of inducing ER‐stress, A375 cells were treated with thapsigargin, a drug that induces a lethal ER‐stress response by inhibiting the ATPase that regulates ER calcium homeostasis [43]. Thapsigargin upregulated expression of glycosylated IL24 protein (Fig. 2D). To determine whether or not induction of IL24 expression was linked to its glycosylation, A375 cells were treated with tunicamycin, a drug that induces a lethal ER‐stress response by inhibiting protein glycosylation, thereby preventing proper folding and trafficking to the Golgi [44, 45, 46]. Tunicamycin upregulated expression of unglycosylated IL24 protein (Fig. 2D). Thapsigargin and tunicamycin each induced 3× the amount of *IL24* RNA in melanoma A375 cells as did an equivalent concentration of WX8 (Fig. 2E). Thus, upregulation of IL24 expression in melanoma cells was a consequence of induction of lethal levels of ER‐stress.

### 3.3 PERK‐Dependent ER‐Stress response in melanoma cells

ER‐stress results when excess unfolded proteins activate one or more of the three ER‐stress sensors: EIF2AK3/PERK protein kinase, ERN1/IRE1α inositol‐requiring enzyme, and ATF6 transcription factor [47]. Thapsigargin induces a PERK‐dependent ER‐stress response in which an excess of unfolded proteins activates the PERK kinase via autophosphorylation [43]. This event results in phosphorylation of EIF2A/eIF2α, increased translation of the ATF4/TXREB transcription factor, and upregulation of genes that inhibit cell proliferation and induce cell death (diagram Fig. S2B) [45]. These events were confirmed in melanoma A375 cells.

Thapsigargin induced the time‐dependent phosphorylation of EIF2A protein, followed by upregulation of ATF4 transcription factor and then the stress response transcription factor DDIT3 (Fig. 2G). Thapsigargin did not induce expression of the ATF6‐dependent ER‐stress response protein, although it did induce expression of XBP1s, a component of the IRE1/ERN1‐dependent ER‐stress response [48].

To determine whether or not XBP1 activation was PERK‐dependent or simply thapsigargin dependent, three different inhibitors of the PERK‐dependent ER‐stress response (Fig. S3A) were tested for their ability to prevent thapsigargin induction of ER‐stress response genes under conditions that did not inhibit melanoma A375 proliferation (Fig. S3B). GSK2656157 inhibits PERK activity with an IC_50_ of 0.9 nm [49], and AMG44 inhibits PERK activity with an IC_50_ of 6 nm [50]. Both drugs inhibited thapsigargin induced PERK‐dependent phosphorylation of EIF2A and expression of ATF4 in melanoma A375 cells (Fig. S3C). In contrast, ISRIB increased the cellular concentration of EIF2A‐P comparable to the level induced by thapsigargin (Fig. S3C). ISRIB prevents EIF2A‐P from binding to EIF2B (a guanine nucleotide exchange factor for EIF2) with an IC_50_ of 5 nm, thereby elevating cellular levels of EIF2A‐P with opposite effect on ATF4 translation [51]. Thus, thapsigargin induced a PERK‐dependent ER‐stress response in melanoma cells that included upregulation of ATF4. This event did not require either the ATF6 or the ERN1 activities, because neither CeapinA7, a specific inhibitor of the ATF6 kinase, nor any of four specific inhibitors of ERN1 (STF083010, MKC3946, APY29, or KIRA6) prevented synthesis of XBP1s protein (Fig. S3E). Taken together, these results revealed that the PERK‐dependent ER‐stress response in melanoma cells triggered upregulation of *IL24* gene expression.

### 3.4 WX8 induced the PERK‐Dependent ER‐Stress response in melanoma cells

RNA‐seq profiling (Fig. 2A) revealed that genes in the PERK‐dependent ER‐stress response were significantly upregulated in A375 cells (*P* < 0.001 to < 0.0001) but not in in HFF1 cells (*P* > 0.05). MAPK13/p38d and its downstream targets upregulated the growth arrest and DNA damage inducible genes PPP1R15A/GADD34, GADD45A, and DDIT3, as well as the pro‐apoptotic genes BAK1/CDN1, PMAIP/NOXA, and BBC3/PUMA. These genes induce cell death [52, 53]. The cyclin‐dependent kinase inhibitor CDKN1A/p21 inhibits the cyclin‐dependent kinases required for cell proliferation [54]. Diagramed in Fig. S2B.

**Fig. 3:**
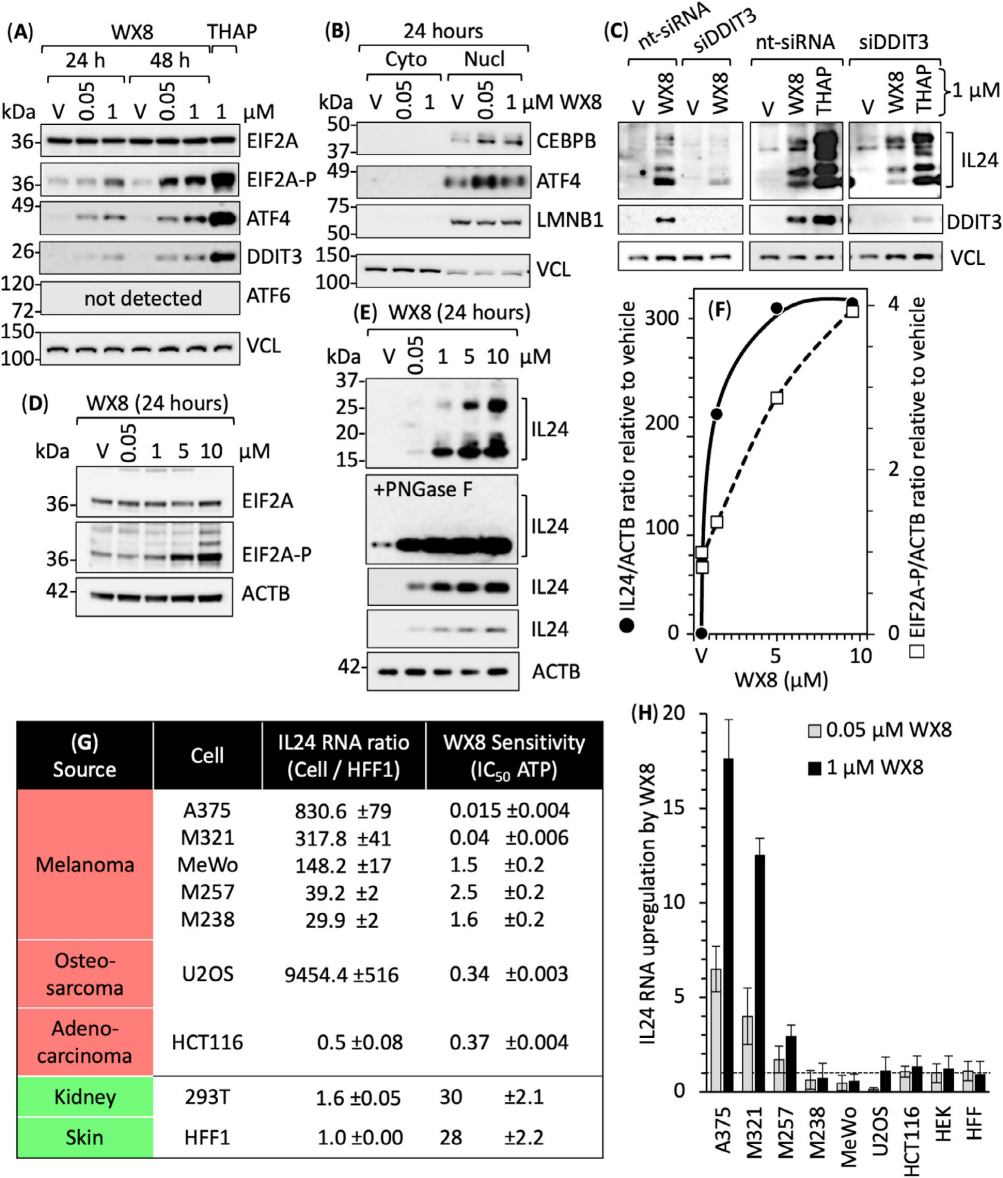
The EIF2AK3/PERK‐dependent ER‐stress response upregulated IL24 expression. (A) Melanoma A375 cells were cultured with either vehicle (V), 0.05 μm WX8, or 1 μm WX8 for either 24 or 48 h. Whole cell extracts were then immuno‐blotted for the indicated proteins, which were identified by their molecular weight and their reaction with a specific antibody. Thapsigargin (THAP) 1 μm was included for comparison. (B) Cells were treated for 24 h with vehicle (V), 0.05 μm WX8 or 1 μm WX8 and the indicated proteins identified by immuno‐blotting from both cytoplasm and nuclear lysate. Lamin B1 (LMNB1) and Vinculin (VCL) were used as nuclear and cytoplasmic control proteins. (C) Cells were treated for 48 h with vehicle (V), 1 μm WX8 or 1 μm THAP, and either nontargeted siRNA (nt‐siRNA) or siRNA targeted against DDIT3 (siDDIT3). The indicated proteins were detected by immunoblotting whole cell extracts. (D) Cells were cultured for 24 h with increasing concentrations of WX8, and the indicated proteins (eIF2α, eIF2α‐P, and ACTB) identified by immunoblotting of whole cell extracts. (E) IL24 protein [both glycosylated and de‐glycosylated (PNGase F treated) were immunoblotted from samples as described in panel D]. (F) The ratios of IL24/ACTB and EIF2A‐P/ACTB were quantified by densitometry from the data in panels D and E. (G) The ratio of IL24 RNA relative to HFF1 IL24 RNA was determined by RT‐PCR in the indicated cell lines (± SEM, *n* = 3) and compared with cell viability, as previously quantified by their IC_50_ for ATP loss [13]. (H) The indicated cell lines were cultured for 24 h with vehicle, 0.05 μm WX8 (gray bar) or 1 μm WX8 (black bar). The ratios of IL24 RNA in WX8‐treated cells to vehicle‐treated cells were quantified by RT‐PCR (±SD, *n* = 2). Images in panels A through F are representative of three samples.

As with thapsigargin, WX8 induced PERK‐dependent phosphorylation of EIF2A protein and upregulation of ATF4 and DDIT3 proteins in melanoma A375 cells (Fig. 3A) accompanied by translocation of transcription factors ATF4 and CEBPb to the nucleus (Fig. 3B). Moreover, EIF2A phosphorylation and IL24 gene expression were WX8 dependent (Fig. 3D–F). Neither WX8 nor thapsigargin upregulated ATF6. In contrast with thapsigargin (Fig. 2G), WX8 did not induce expression of XBP1s (Fig. S3C,D). WX8 induced only the PERK‐dependent ER‐stress response in melanoma cells.

### 3.5 IL24 upregulation depended on the DDIT3 transcription factor

*IL24* gene expression is dependent on transcription factors CEBPb, JUN, and FOS [55, 56]. Transcription of all three was upregulated significantly by WX8 in A375 but not HFF1 cells (Fig. 2A). Both CEBPb and JUN proteins were significantly upregulated in nuclear extracts of A375 but not in HFF1 (Fig. S1B), consistent with upregulation of IL24 expression in A375 but not HFF1 cells (Figs 1 and 2B).

DDIT3/CHOP is also a C/EBP transcription factor termed CEBPz [57]. WX8 significantly upregulated DDIT3 RNA and protein in A375 cells (Figs 2A and 3C), and siRNA targeted against DDIT3 (siDDIT3) suppressed DDIT3 protein expression and induction of IL24 by WX8 (Fig. 3C). Similar results were obtained with thapsigargin (Figs 2G and 3C). Thus, PERK‐dependent ER‐stress provides a feedback loop that upregulates *IL24* gene expression by DDIT3 (Fig. S2B).

### 3.6 IL24 upregulation was triggered specifically by PIKFYVE inhibition

To determine whether or not upregulation of *IL24* gene expression linked to inhibition of PIKFYVE, *IL24* RNA in A375 cells was quantified following treatment with various metabolic inhibitors. WX8 [14], apilimod [58], Vacuolin‐1 [59], and YM201636 [60] are PIKFYVE inhibitors from three different chemical groups [fig. S1 in ref. 13]. Chloroquine disrupts autophagy by decreasing lysosomal acidity, thereby rendering pH‐dependent lysosomal hydrolases nonfunctional [61]. Bafilomycin‐A1 disrupts autophagy by inhibiting the vacuolar ATPase required for lysosome acidification [62, 63]. Adriamycin and etoposide induce double‐strand DNA breaks [64].

**Fig. 4:**
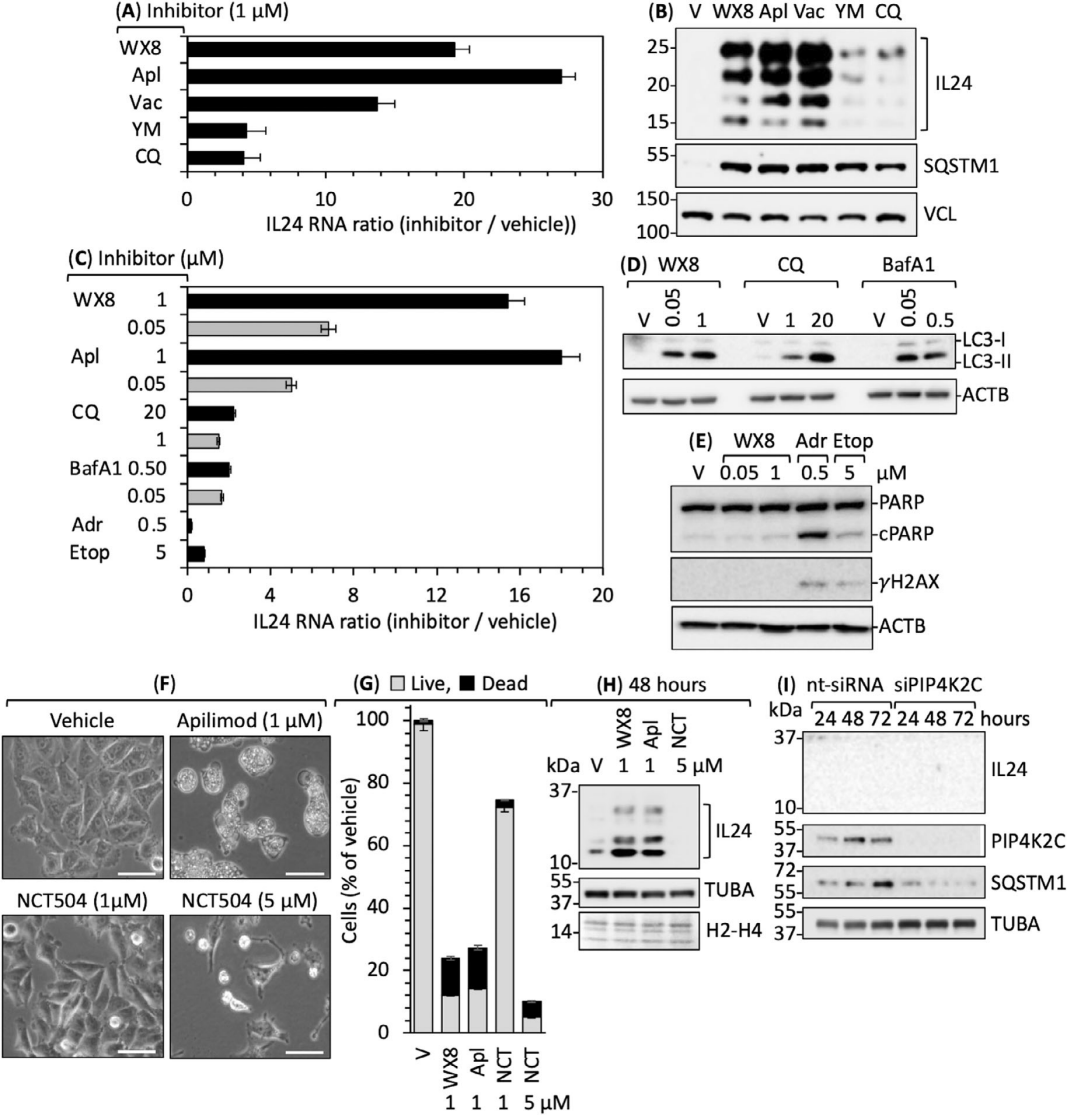
IL24 upregulation was specific for PIKFYVE inhibition. (A) Melanoma A375 cells were cultured with the indicated PIKFYVE inhibitor (WX8, Apilimod/Apl, Vacuolin‐1/Vac, YM201636/YM) or autophagy inhibitor (Chloroquine/CQ) for 24 h. Expression of IL24 RNA relative to vehicle was quantified using RT‐PCR. (B) Example of an IL24 immunoblot from panel A in which IL24 protein is visible for all five inhibitors. (C) A375 cells were cultured for 24 h with the indicated inhibitors [WX8, Apl, CQ, Bafilomycin‐A1 (BafA1), Adriamycin (Adr), etoposide (Etop)]. The ratio of IL24 RNA in cells treated with inhibitor to RNA in cells treated with vehicle was quantified by RT‐PCR. (D) A375 cells were cultured for 24 h with the indicated concentrations of WX8, CQ and BafA1. The relative amounts of LC3‐II protein were detected by immunoblotting of whole cell extracts, thereby confirming that each inhibitor blocked autophagic flux to an equivalent extent. (E) A375 cells were cultured for 24 h with either vehicle (V), or the indicated concentration of WX8, Adr, or Etop. Equal amounts of total cell extract were subjected to immunoblotting for PARP, cleaved PARP (c‐PARP), γH2AX, and the cytoplasmic protein ACTB. Only Adr and Etop induced DNA damage, as indicated by the appearance of cPARP and γH2AX. (F) Phase contrast images of A375 cells treated with vehicle, apilimod, or NCT‐504 for 48 h at the indicated concentration. Bar indicates 20 μm. (G) Percentage of live (black) and dead A375 cells (gray) after treatment of vehicle, WX8, apilimod, and NCT‐504 for 72 h. (H) A375 cells are treated with indicated concentrations of WX8, apilimod, and NCT‐504 and IL24 protein was detected through immunoblotting. (I) A375 cells are treated with either nt‐siRNA or PIP4K2C siRNA for 24–72 h and cell lysates were immunoblotted for IL24. Error bars in panels A, C and G are ±SD, *n* = 2. Panels B, D–F, H, and I are representative of three samples.

WX8, apilimod, and Vacuolin‐1 each induced robust upregulation of IL24 RNA and protein levels relative to chloroquine (Fig. 4A–C). Notably, YM201636 did not. Thus, despite the fact that WX8, chloroquine and bafilomycin‐A1 disrupted autophagic flux under these conditions (Fig. 4D), only the three strongest PIKFYVE inhibitors induced a robust increase in *IL24* expression.

Upregulation of *IL24* expression was not a response to DNA damage because WX8 did not induce DNA damage (Fig. 4E) under conditions where it upregulated *IL24* expression by 18X (Fig. 2B,C). Moreover, neither Adriamycin nor etoposide induced *IL24* expression under conditions where they did induced DNA damage (Fig. 4E), as confirmed by cleavage of poly(ADP‐ribose) polymerase (PARP) and expression of gH2AX [65].

Upregulation of *IL24* expression did not result from inhibition of PIP4K2C. At low concentrations, WX8 specifically inhibits PIKFYVE *in situ*, whereas at higher concentrations WX8 inhibits both PIKFYVE and PIP4K2C, thereby dramatically suppressing PI(4,5)P_2_ synthesis in autophagy‐dependent cells that are deficient in PI5K1C [13]. These cells require PIP4K2C to convert PI5P into PI(4,5)P_2_, a phosphoinositide essential for macro‐autophagy [2, 15, 16]. Consequently, selective inhibition of PIKFYVE alone inhibits cell proliferation, whereas inhibition of both PIKFYVE and PIP4K2C, either with 1 μm WX8 or with 0.05 μm WX8 together with siRNA against PIP4K2C, induces cell death [13].

To determine whether or not upregulation of IL24 expression required inhibition of both PIKFYVE and PIP4K2C, melanoma A375 cells were treated with either vehicle, WX8, apilimod, or NCT504, a specific inhibitor of PIP4K2C [66]. Concentrations of these inhibitors that arrested cell proliferation and induced cell death within 48 h (Fig. 4F,G) were then tested for their ability to induce expression of IL24 protein (Fig. 4H). WX8 and apilimod induced IL24, but NCT504 did not. This result was confirmed using siRNA targeted against PIP4K2C (Fig. 4I). Thus, PIKFYVE activity prevented over‐expression of IL24 in melanoma cells, whereas PIP4K2C did not.

### 3.7 Upregulation of IL24 was limited to melanoma cells

To determine whether or not WX8 mediated induction of IL24 was cell type specific, nine cell lines representing two normal tissues and three cancers (Fig. 3G) were cultured for 24 h with either vehicle or WX8 (Fig. 3H). Their basal levels of IL24 RNA relative to HFF1 varied 18 900 fold. Their sensitivity to WX8, as quantified by the IC_50_ for ATP loss, varied 2000‐fold [13]. Induction of IL24 expression by 1 μm WX8 varied from null to 18× above vehicle (Fig. 3H). Three of the five melanoma lines tested were sensitive to increasing concentrations of WX8, and the two most sensitive lines also had the highest basal levels of IL24. Thus, melanoma cells that produce high levels of IL24 are most likely to be sensitive to PIKFYVE inhibition.

In contrast with melanoma, osteosarcoma U2OS cells had the highest basal level of IL24 RNA and adenocarcinoma HCT116 had the lowest, but their sensitivity to WX8 was the same. In contrast to the three cancers tested, nonmalignant cells had the lowest basal levels of IL24 RNA, and they were the least sensitive to WX8.

### 3.8 WX8 inhibited formation of melanoma tumors

**Fig. 5:**
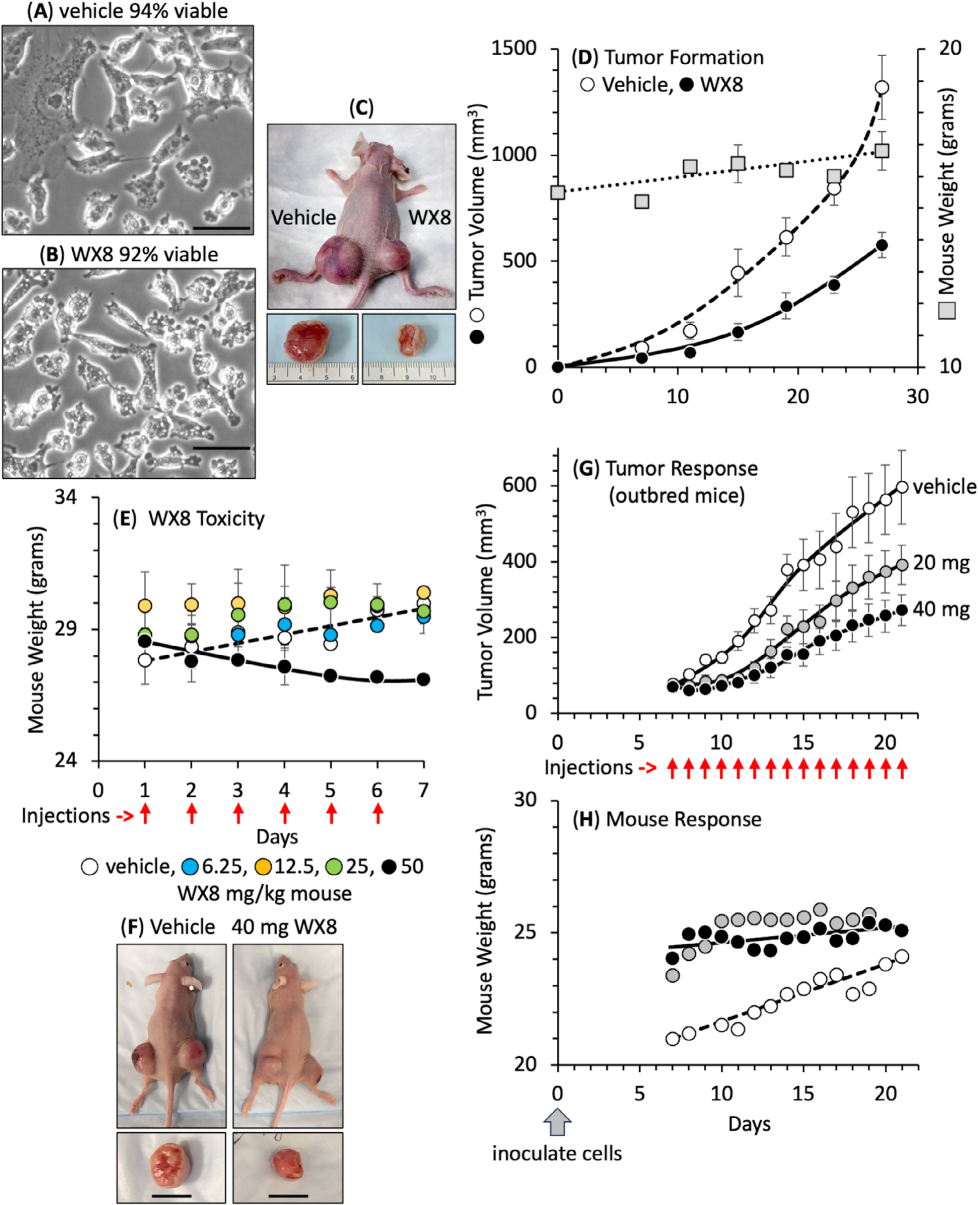
WX8 inhibited formation and growth of melanoma tumors. Melanoma A375 cells were cultured for 8 h with either vehicle (A) or 10 μm WX8 (B) to inhibit PIKFYVE effectively (extensive cytoplasmic vacuolation) while retaining cell viability (resistance to trypan blue). Bar indicates 20 μm. Cytoplasmic vacuolization occurred only in the WX8‐treated cells. (C) Nude mice (*n* = 5) were immediately inoculated in their left flank with cells pretreated with vehicle and in their right flank with cells pretreated with WX8. Tumors were excised at 28 days. Ruler is in cm. (D) Periodic measurements of tumor volume and mouse weight revealed that cells pretreated with vehicle (open circles) formed tumors more rapidly than cells pretreated with WX8 (solid circles). Mouse weight gradually increased (shaded squares, ± SEM, *n* = 5). (E) To evaluate WX8 toxicity, CD1 IGS mice received daily intraperitoneal injections of either vehicle or WX8. Mice were weighed each day (±SD, *n* = 2). (F) Outbred homozygous nude mice were inoculated on both flanks with melanoma A375 cells and then palpable tumors were allowed to form. Tumors were excised at 21 days. Bar indicates 1 cm. (G) Mice with palpable tumors received daily intraperitoneal injections of either vehicle or WX8 and tumor volume quantified. (H) Mice in panel G were weighed each day (± SEM, *n* = 3, for each treatment). Images in panels A–C are representative of three samples.

To determine whether or not WX8 could inhibit progression of a tumor that arose from PIKFYVE‐sensitive cancer cells, melanoma A375 cells were cultured for 8 h with either vehicle or 10 μm WX8. Cells attached to the dish were then isolated by trypsinization, and a sample stained with trypan blue. Cells treated with vehicle were 94% viable (excluded trypan blue) and displayed no cytoplasmic vacuolation (Fig. 5A). Cells treated briefly with WX8 displayed cytoplasmic vacuolation but remained viable (Fig. 5B). Immune‐compromised inbred mice were inoculated subcutaneously with vehicle‐treated cells on one flank and WX8‐treated cells on the other flank (Fig. 5C). The WX8‐treated cells produced tumors more slowly than vehicle‐treated cells (Fig. 5D). Thus, inhibition of PIKFYVE inhibited PIKFYVE‐sensitive melanoma cells from forming a tumor.

### 3.9 WX8 inhibited expansion of melanoma tumors

To determine whether or not WX8 could inhibit expansion of preformed tumors that arose from PIKFYVE‐sensitive cells, melanoma A375 cells were inoculated into the flanks of immuno‐compromised mice and allowed to form palpable tumors. Mice were then injected intraperitoneally once a day with either vehicle or nontoxic levels of WX8. Toxicity was determined by injecting increasing amounts of WX8 in wild‐type mice (Fig. 5E). The result revealed a modest decrease in bodyweight at 50 mg WX8 per kg mouse body weight over a period of 7 days. Therefore, 20 and 40 mg WX8·kg^−1^ were used to determine the effect of WX8 on preformed melanoma tumors.

**Fig. 6:**
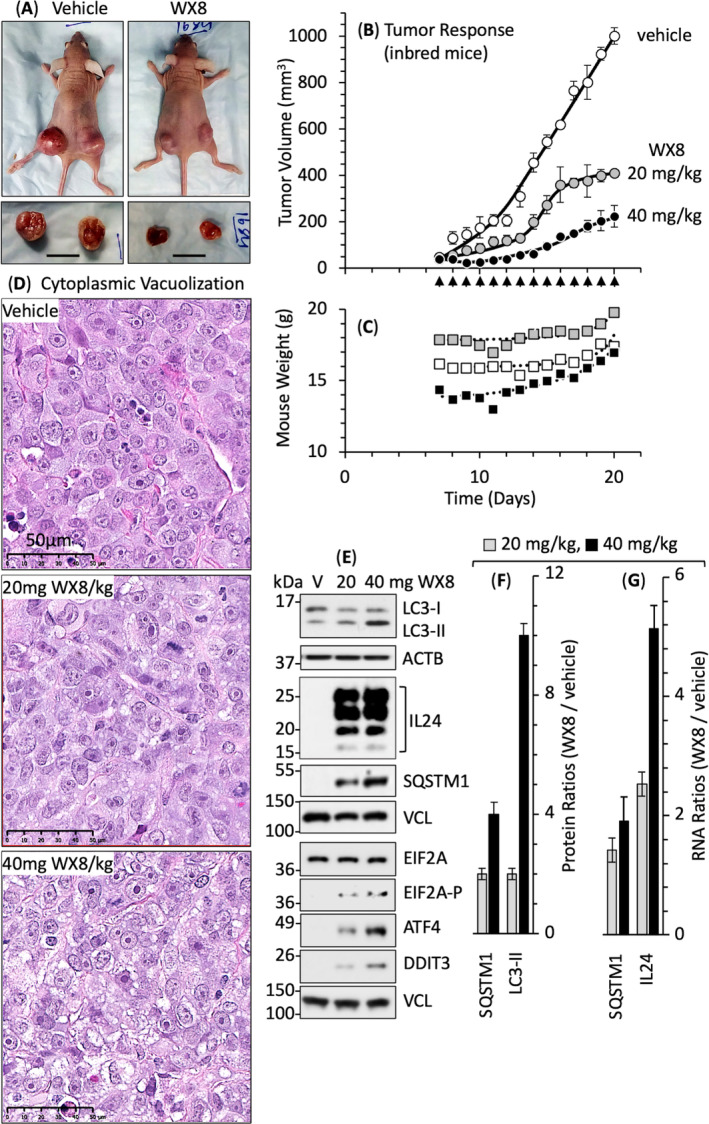
WX8 disrupted autophagy and upregulated IL24 gene expression in melanoma A375 tumors. (A) Inbred female BALB/c nu/nu mice were inoculated on both flanks with melanoma A375 cells. Tumors were excised 21 days postinoculation. Bar indicates 1.5 cm. (B) When palpable tumors were evident (day 6), intraperitoneal injections of either vehicle (open circles), 20 mg WX8·kg^−1^ mouse weight (shaded circles), or 40 mg WX8·kg^−1^ mouse weight (solid circles) were administered each day (arrows) for 14 days. Tumor growth was inhibited in proportion to the WX8 concentration. Three mice were used for each treatment group. (C) Mouse body weights increased with time. (D) Tumors were excised at 21 days postinoculation and tumor sections were stained with hematoxylin and eosin. Vacuolation surrounding nuclei was evident in tumors from WX8‐treated mice but absent in vehicle‐treated mice. Bar indicates 50 μm. (E) Extracts from tumor slices revealed WX8‐dependent accumulation of LC3‐II, IL24, SQSTM1/p62, EIF2A, EIF2A‐P, ATF4, and DDIT3 proteins in tumors from WX8‐treated mice. ACTB and VCL are cytoplasmic proteins. Phosphorylated proteins are unstable in frozen samples. (F) SQSTM1 and LC3‐II proteins in panel E were quantified (±SD, *n* = 2). (G) SQSTM1 and IL24 RNA abundance from tumors from WX8‐treated mice relative to tumors in vehicle‐treated mice was quantified by RT‐PCR (± SEM, *n* = 3 mice or three tumors). Images in panels A, D, and E are representative of three samples.

Outbred mice represent an undefined genetic complexity that reveals the maximum variation in drug efficacy, whereas inbred mice reveal the minimum variation. In outbred mice, injections of up to 40 mg WX8·kg^−1^ mouse inhibited tumor expansion (Fig. 5F,G) without affecting either mouse body weight or behavior (Fig. 5H). Similar results were obtained with inbred mice (Fig. 6A) but with significantly less variation from one mouse to another (Fig. 6B,C).

### 3.10 WX8 induced the same effects in tumors as in cells

WX8 disrupted lysosome homeostasis in melanoma tumors, as evidenced by WX8 dose‐dependent cytoplasmic vacuolation in tumors from WX8‐treated mice but absent in tumors from vehicle‐treated mice (Fig. 6D). WX8 disrupted autophagy in melanoma tumors, as evidenced by increased levels of LC3‐II and SQSTM1 proteins and RNA (Fig. 6E–G). WX8 also induced the IL24 ER‐stress response in these tumors, as evidenced by upregulation of IL24 RNA and protein (Fig. 6E,G) as well as phosphorylation of EIF2A and upregulation of ATF4 and DDIT3 (Fig. 6E). Thus, WX8 exhibited therapeutic potential in the treatment of PIKFYVE‐sensitive tumors by inducing the same biochemical changes in tumors as it did in cultured cells.

### 3.11 Ectopic expression of IL24 protein selectively induced melanoma cell death

**Fig. 7:**
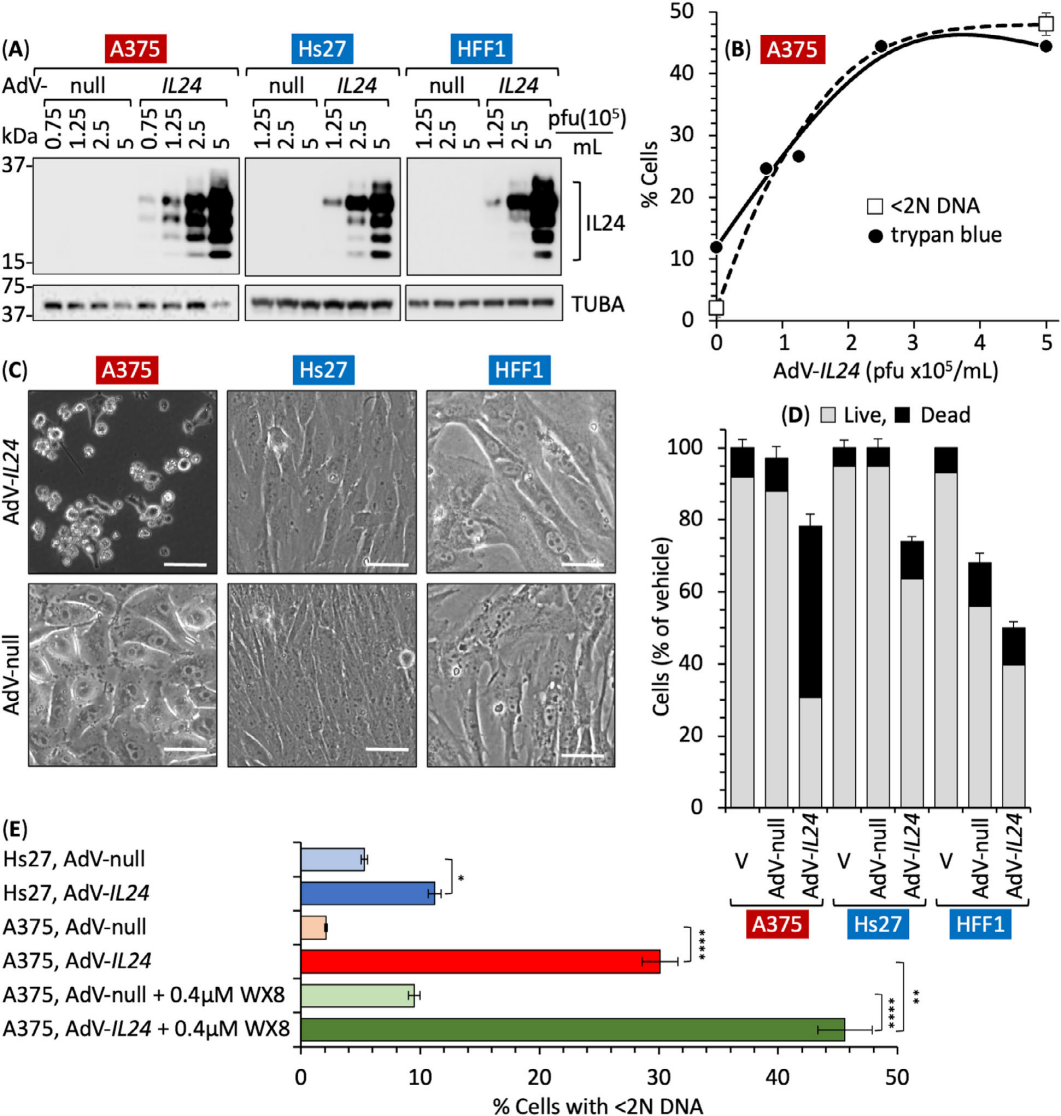
Ectopic expression of IL24 protein selectively induced melanoma cell death. (A) Melanoma A375 and foreskin fibroblasts Hs27 and HFF1 were infected for 72 h with the indicated number of plaques forming units (pfu) per mL of either AdV‐null or AdV‐*IL24*. Total cell extracts were then immunoblotted for IL24 protein. (B) The fraction of dead cells was determined at different pfu concentrations by staining one group with trypan blue and a separate group with propidium iodide. FACS analysis revealed the fraction of cells with < 2N DNA (Fig. S4). (C) Phase contrast images of cells infected with either AdV‐null or AdV‐*IL24* with 5 × 10^5^ pfu·mL^−1^. Bar represents 20 μm. (D) Attached cells were collected by trypsinization, combined with unattached cells in culture medium, stained with trypan blue and then quantified as either live or dead for A375, Hs27 and HFF1 cell lines infected with either Adv‐Null or Adv‐*IL24*. (E) The indicated cell populations were subjected to FACS analysis to quantify the faction of cells with < 2N DNA content (Fig. S4). A375 cells were also cultured with 0.4 μm WX8 ± either AdV‐null or AdV‐*IL24* for comparison. Panels B, D, and E include ±SD, *n* = 2. Statistical significance was *P* < 0.05 (*), *P* < 0.01 (**), *P* < 0.0001 (****). *P*‐values in RNA‐seq were determined by Wald Chi‐square test using the DEseq2 platform. *P*‐values of two data sets from the other experiments were calculated by Student's paired two‐tailed *t*‐test using graphpad prism. Images in panels A and C are representative of three samples.

Previous studies have reported that ectopic expression of IL24 can selectively terminate cancer cells with little, if any, effect on normal cells [25, 26, 27, 28, 67]. Therefore, to determine whether or not ectopic IL24 would selectively terminate the melanoma cells used herein, PIKFYVE‐sensitive melanoma A375 and PIKFYVE‐insensitive human HFF1 and Hs27 foreskin fibroblasts [7] were infected with the same adenovirus (AdV) derived IL24 expression vectors used in previous studies. Only cells infected with AdV‐*IL24* expressed ectopic glycosylated forms of the IL24 protein; cells infected with AdV‐null did not (Fig. 7A). The fraction of dead cells (indicated by permeability to trypan blue) was confirmed by FACS analysis to quantify the fraction of cells with < 2N DNA (Fig. S4). The fraction of dead cells increased in proportion to the concentration of virus used (Fig. 7B). The maximum number of dead A375 cells (~ 45%) was achieved with 3–5 × 10^5^ pfu·mL^−1^.

Ectopic expression of IL24 in A375, Hs27, and HFF1 cells suppressed their proliferation, thereby reducing the total number of cells present after 72 h of culture (Fig. 7D). However, of the cells present, 53% of A375 cells were killed (trypan blue positive) by AdV‐*IL24*, whereas only 9% of Hs27 and 11% of HFF1 cells were killed (Fig. 7D). Moreover, AdV‐*IL24* significantly altered the morphology of the remaining melanoma cells, but not the remaining fibroblasts (Fig. 7C), and increased the fraction of melanoma dead cells (< 2N DNA content) 15‐fold but the fraction of fibroblasts only two‐fold (Fig. 7D).

The effect of ectopic expression of IL24 together with inhibition of PIKFYVE by WX8 was additive (Fig. 7E). AdV‐*IL24* induced death in 30% of A375 cells, and 0.4 μm WX8 induced death in 10% of A375 cells. In combination, they induced death in 45% of A375 cells, thereby confirming that cell death was linked directly to over‐expression of IL24.

### 3.12 Suppression of *IL24* expression reduced sensitivity to PIKFYVE inhibition

To determine whether or not IL24 was essential for WX8 inhibition of either cell proliferation or induction of cell death, melanoma A375 cells were treated with siRNA targeted against IL24 (siIL24). Nontarget (nt) siRNA was included as a control. Expression of IL24 protein was strongly suppressed by siIL24 (Fig. S5A) with no apparent change in cell morphology (Fig. S5B). However, siIL24 increased the IC_50_ for WX8 suppression of cell proliferation approximately four‐fold without significantly reducing viability (Fig. S5C). Nevertheless, siIL24 did reduce the fraction of dead A375 cells within the total cell population (Fig. S5D).

These results were consistent with the fact that the endogenous level of IL24 RNA in melanoma cells was at least 800× the level in WX8‐resistant cells (Fig. 3G). Therefore, even a 90% reduction in the endogenous level of endogenous IL24 protein would have little effect on melanoma viability.

### 3.13 IL24 was essential for WX8 induction of cell death

**Fig. 8:**
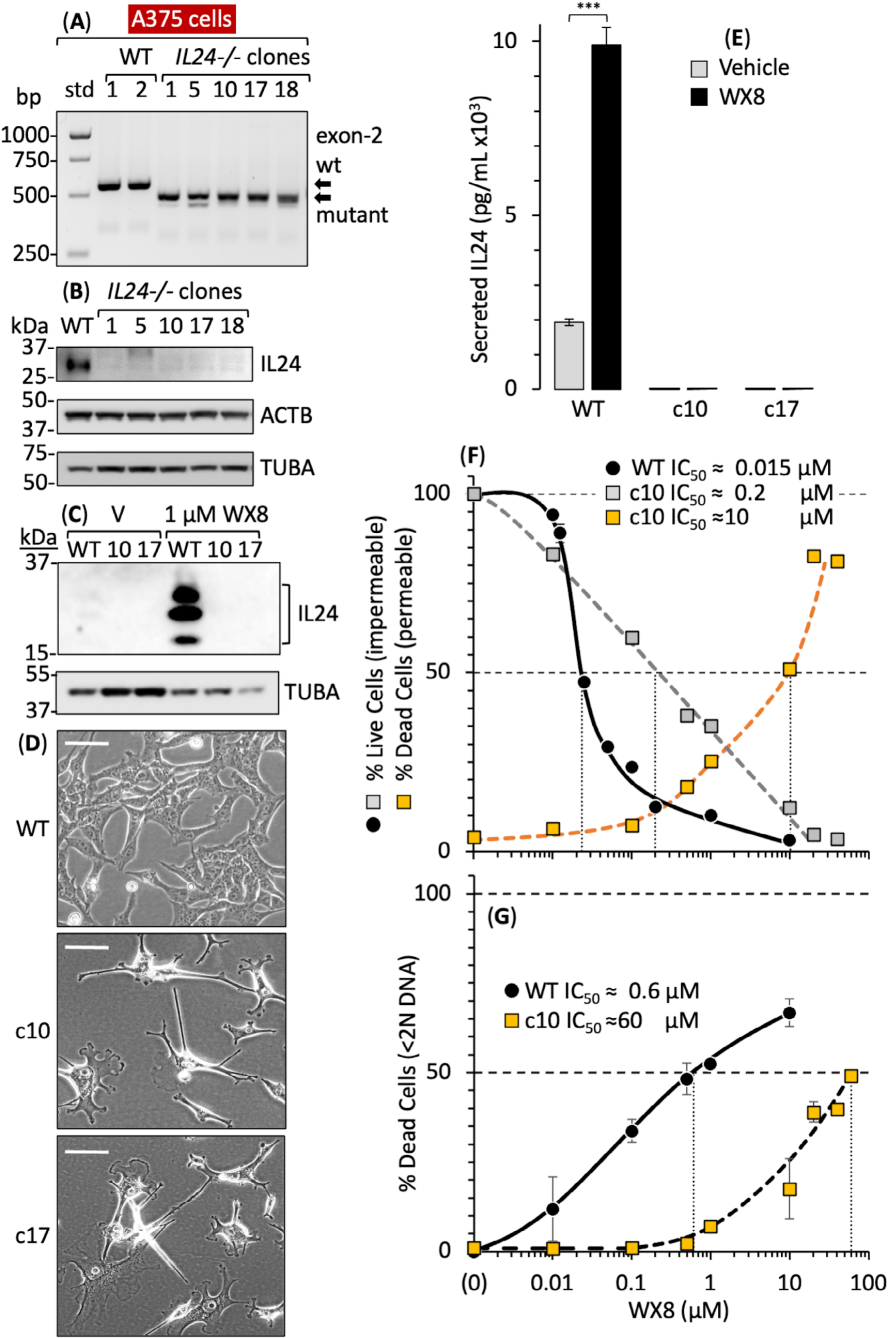
*Il24* gene expression is essential for WX8‐dependent cell death. Exon‐2 in the *IL24* gene was ablated in melanoma A375 cells. (A) Surviving clones were isolated and screened for deletion within exon‐2 of *IL24* gene and comparison with DNA size standards (std) using PCR technology. (B) Immunoblot of total cell extracts confirmed the absence of IL24 protein in A375(*IL24* ^*−/−*^) clones. (C) WX8 upregulated expression of IL24 protein in wild‐type A375 cells but not in A375(*IL24* ^*−/−*^) clones exemplified by c10 and c17. (D) Phase contrast images of A375 and A375(*IL24* ^*−/−*^) cells. Bar indicates 20 μm. (E) ELISA assays confirmed the absence of IL24 protein in the cell culture medium of A375(*IL24* ^*−/−*^) clones cultured for 24 h in the presence of either vehicle or 1 μm WX8. (F) The fractions of live (impermeable to trypan blue) and dead (permeable to trypan blue) A375 and A375(*IL24* ^*−/−*^) cells after culturing A375 wild‐type and A375(*IL24* ^*−/−*^) clone 10 cells for 72 h with the indicated concentrations of WX8. (G) The fraction of cells with < 2N DNA content (less DNA than G1 phase cells) were considered dead. To allow a logarithmic axis, vehicle was plotted as 0.001 μm WX8. Panels E–G include ±SD, *n* = 2. *P*‐values in RNA‐seq were determined by Wald Chi‐square test using the DEseq2 platform. *P*‐values of two data sets from the other experiments were calculated by Student's paired two‐tailed *t*‐test using graphpad prism. Images in panels A through D are representative of three samples.

To determine whether or not IL24 was essential for WX8 to induce cell death, *IL24* exon2 was ablated in melanoma A375 cells using CRISPR‐Cas9 technology, and viable A375(*IL24* ^−*/−*^) clones were isolated (Fig. 8A). They contained a homozygous deletion of 74 bp within *IL24* exon2. Consequently, IL24 protein was not detected in any of the *IL24* ^−*/−*^ clones (Fig. 8B) and culturing them with WX8 did not induce expression of either endogenous (Fig. 8C) or secreted (Fig. 8E) IL24 protein. In contrast with siIL24‐treated cells, A375(*IL24* ^*−/−*^) cells proliferated slowly and exhibited abnormally long projections characteristic of cytoskeletal actin projections that occur at the leading edge of migratory cells [68, 69, 70] (Fig. 8D).

To determine the effect of ablating IL24 genes in melanoma cells on their sensitivity to WX8, A375(WT) and A375(*IL24* ^*−/−*^) cells were cultured 72 h in the presence of either vehicle or increasing concentrations of WX8. Cells were then stained with trypan blue to distinguish live cells (unstained) from dead cells (stained). The IC_50_ for WX8 inhibition of cell proliferation was quantified by the fraction of live cells (Fig. 8F). Proliferation of WT A375 cells was 13× more sensitive to WX8 than was proliferation of A375(*IL24* ^*−/−*^) cells, and permeabilization of the cell membrane was 50× less sensitive to WX8 than cell proliferation.

The IC_50_ for WX8 induction of cell death was quantified by the fraction of cells with a DNA content less than 2N, the amount in G1 phase cells (Fig. 8G). Induction of death was 100X more sensitive to WX8 in WT A375 cells than in A375(*IL24* ^*−/−*^) cells. Comparing trypan blue staining with DNA loss revealed that cell permeability was 6× more sensitive to WX8 than was DNA loss and cell death.

As previously reported, PIKFYVE inhibitors induce noncanonical apoptosis selectively in PIKFYVE‐sensitive cells [4, 7, 9, 13]. Loss of DNA in A375 cells was 70× more sensitive to WX8 than HFF1 cells (Fig. S6A), and 1 μm WX8 induced DNA damage in A375 cells under conditions where it did not in HFF1 cells (Fig. S6B). However, it did not induce cleavage of caspase‐3, and WX8‐induction of cell death was not prevented by inhibiting caspase activity, consistent with noncanonical apoptosis (Fig. S6C,D).

## 4 Discussion

The results reported here reveal the mechanism by which PIFKVYE inhibitors induce cell death selectively in PIKFYVE‐sensitive melanoma cells and tumors without harming the viability of normal cells. PIKFYVE inhibitors disrupt lysosome homeostasis, which resulted in ER‐stress in melanoma cells and tumors but not in normal foreskin fibroblasts. ER‐stress induced the PERK‐dependent ER‐stress response that includes upregulation of DDIT3/CHOP/CEBPz, a transcription factor that promotes expression of genes, such as IL24, that require a C/EBP family transcription factor. The fact that PIKYFVE specific inhibitors, but not PIP4K2C‐specific inhibitors, induced expression of the IL24 gene suggests that PI(3,5)P2 or PI5P or both are involved in regulating IL24 transcription.

Upregulation of *IL24* expression played a minor role in the ability of ER‐stress to inhibit cell proliferation, but it was essential to induce cell death. Low levels of WX8 specifically inhibit PIKFYVE thereby upregulating IL24 expression and suppressing cell proliferation, whereas higher concentrations further upregulate IL24 and inhibit PIK4K2C, the downstream phosphoinositide kinase required to convert the phosphoinositides produced by PIKFYVE into PI(4,5)P_2_, a phosphoinositide required for macro‐autophagy [13]. Since selective inhibition of PIK4K2C did not upregulate IL24 expression, melanoma cell death involves two events: increasing ER‐stress by over‐expression of a secreted glycosylated protein and suppressing synthesis of PI(4,5)P_2_.

### 4.1 Cell proliferation

Induction of an ER‐stress response in autophagy‐dependent cancer cells inhibits cell proliferation by upregulating the transcription factor ATF4. This event upregulates expression of CDKN1A/p21, which inhibits cyclin‐dependent kinases CDK1 and CDK2. Since these enzymes are essential for proliferation of all mammalian cells, inhibition of PIKFYVE reduces the rate of proliferation in nonmalignant as well as malignant cells.

Selective inhibition of PIKFYVE induces a characteristic cytoplasmic vacuolation and inhibits cell proliferation in mammalian cells [11, 13]. However, suppression of IL24 expression with siRNA only marginally reduced proliferation of either melanoma A375 cells or HFF1 foreskin fibroblasts. Remarkably, ablation of the IL24 gene reduced A375 proliferation and altered its morphology, suggesting a role for IL24 during terminal cell differentiation [71, 72]. In addition, WX8 was 13× less effective at inhibiting proliferation of A375(*IL24* ^*−/−*^) cells than with A375 cells. Hence, melanoma cells require some basal level of *IL24* gene expression to support proliferation.

### 4.2 Cell death

ER‐stress results when the capacity of the ER to fold proteins becomes saturated, a phenomenon triggered by impaired protein glycosylation, disulfide bond formation, or overexpression of secreted proteins [42, 47, 73]. ER‐stress initially attenuates protein synthesis and upregulates protein chaperones to promote the processing and refolding of proteins. If the accumulation of unfolded proteins is not relieved, then cell death occurs. Thus, ectopic expression of IL24 induces a time and dose‐dependent increase in expression of growth arrest and DNA damage‐inducible genes characteristic of PERK‐dependent ER‐stress with concomitant induction of apoptosis [24, 25, 26, 27, 28]. Consistent with these reports, ectopic expression of IL24 in human melanoma cells inhibited proliferation and induced death, whereas ectopic expression of IL24 in normal human fibroblasts did not.

PIKFYVE inhibitors induced ER‐stress by disrupting lysosome homeostasis, and ER‐stress elevated IL24 levels via a feedback loop in which the transcription factor DDIT3/CHOP upregulated *IL24* gene expression, thereby inducing cell death. A375(*IL* ^*−/−*^) cells were resistant to WX8 induction of cell death, but sensitive to WX8 inhibition of cell proliferation. IL24 expression was also upregulated by either thapsigargin or tunicamycin, drugs that induce a lethal ER‐stress response by totally different mechanisms, thereby confirming that IL24 dependent termination of autophagy‐dependent melanoma results from induction of ER‐stress.

Cells that are not autophagy‐dependent, such as human foreskin fibroblasts, are resistant to PIKFYVE inhibitors for two reasons. First, PIKFYVE inhibitors do not upregulate expression of IL24. Second, synthesis of PI(4,5)P_2_ occurs by an alternate pathway. Thus, PIKFYVE‐resistant cells do not experience levels of ER‐stress from PIKFYVE inhibition that trigger a cell death response.

### 4.3 Therapeutic potential

The fact that pre‐exposure of autophagy‐dependent melanoma cells to WX8 suppressed their ability to form tumors in mice revealed a hysteresis in their recovery from PIKFYVE inhibition. The fact that daily intraperitoneal injections of WX8 into mice arrested human melanoma tumors from expanding without visibly affecting the mice demonstrated the therapeutic potential of WX8 in humans. Subcutaneous injection might allow administration of higher drug concentrations. Melanoma sensitivity to PIKFYVE inhibitors was related directly to IL24 expression.

At least 30% of the melanoma cell lines tested were PIKFYVE‐sensitive [13], and the cell lines that were most sensitive to WX8 also upregulated IL24 expression the most. The average levels of IL24 RNA in patient derived melanomas are significantly greater than in samples of normal skin (Fig. S2C), suggesting that PIKFYVE inhibitors alone will have therapeutic potential against many autophagy‐dependent melanomas. Moreover, WX8 together with AdV‐*IL24* overexpression was more than twice as effective in terminating PIKFYVE‐sensitive melanoma cells as either agent alone. Accordingly, PIKFYVE inhibitors could complement the ability of ectopically expressed IL24 to induce cancer‐specific cell death in patients with advanced cancers (discussed in [74, 75]).

Unlike thapsigargin, tunicamycin, and nonsteroidal anti‐inflammatory drugs that induce IL24 ER‐stress indiscriminately, PIKFYVE inhibitors selectively trigger cell death in melanoma cells The nonsteroidal anti‐inflammatory drug sulindac sulfide is a reversible inhibitor of the ER Ca^2+^ ATPase that triggers IL24 ER‐stress via the same mechanism as thapsigargin [76, 77]. Unfortunately, thapsigargin is highly toxic to normal mammalian cells [78], and sulindac alters the status of transcription factors that affect genes involved in cell viability [79, 80]. Nevertheless, sulindac can induce apoptosis in cell lines derived from prostate cancer or head and neck squamous cell carcinoma and inhibit the growth of tumors derived from these cells [77, 81].

Another promising target is colorectal cancer. Lower levels of IL24 in colorectal cancer tissues from patients are associated with lower survival rates [82], and colorectal carcinoma cells are sensitive to ectopic expression of IL24 [83]. Downregulation of KRAS enhanced the ability of ectopic IL24 to suppress proliferation and induce apoptosis in mutant KRAS colorectal cancer cells but not in wild‐type KRAS cells [84]. Colorectal cancer cells such as HCT116 and SW480 are also PIKFYVE‐sensitive [13, 14].

Combinational therapy with inhibitors of the p38 mitogen‐activated protein kinase (p38 MAPK) and PIKFYVE‐inhibitors also appear promising. p38 MAPK inhibitors can suppress cell proliferation and induce apoptosis in melanoma A375 cells, as well as colon cancer HCT116 and SW480 cells [7, 85, 86, 87], cell lines that are PIKFYVE‐sensitive [13, 14] and contain significantly lower levels of p38 MAPK and p38 MAPK phosphorylation than nonmalignant cells [7]. Thus, the combination of WX8 and p38 MAPK inhibitor SB202190 can synergistically disrupt autophagy in SW480 cells and SW480‐derived tumors with little if any harm to nonmalignant cells [7].

## 5 Conclusion

Inhibition of PIKFYVE activity in autophagy‐dependent melanoma cells and tumors upregulates IL24 expression with concomitant amplification of the PERK‐dependent ER‐stress response. This results in cell death and termination of tumor expansion. Thus, PIKFYVE inhibitors, either alone or in combination with other targeted drugs (including ectopic IL24 protein) exhibit significant therapeutic potential against PIKFYVE‐sensitive cancers, particularly melanoma that express high levels of endogenous IL24.

## Conflict of interest

AR and MLD are inventors on U.S. Patent 11471460B2 “Autophagy Modulators for Use in Treating Cancer”.

## Author contributions

AR and ARC executed the experiments. AR and MLD designed the experiments and wrote the manuscript. MLD provided lab space, equipment, and supplies.

### Peer review

The peer review history for this article is available at https://www.webofscience.com/api/gateway/wos/peer‐review/10.1002/1878‐0261.13607.

## Supporting information


**Fig. S1.** Upregulation of genes linked to autophagosomes and lysosomes in melanoma A375 cells and HFF1 foreskin fibroblasts.
**Fig. S2.** WX8 selectively disrupted macro‐autophagy in autophagy‐dependent cells.
**Fig. S3.** Inhibitors of ER‐stress responses and their effect on melanoma A375 cell proliferation.
**Fig. S4.** Induction of cell death by ectopic expression of IL24 was confirmed by accumulation of cells with less than normal amounts of DNA in G1 phase cells.
**Fig. S5.** siRNA suppression of IL24 expression marginally reduced the sensitivity of melanoma A375 to WX8.
**Fig. S6.** WX8‐induced noncanonical apoptosis in melanoma A375 cells.

## Data Availability

All data related to this study are presented in the figures and supplemental figures. The original RNA‐Seq data for A375 and HFF1 cells are available upon request.
