# Supplementary material for: PIKFYVE inhibitors trigger interleukin‐24‐dependent cell death of autophagy‐dependent melanoma
Source: Mol Oncol. 2024 Feb 27;18(4):988–1011. doi: 10.1002/1878-0261.13607 (PMC10994231; doi:10.1002/1878-0261.13607)
Supplement: Supplementary file 1 — Fig. S1. Upregulation of genes linked to autophagosomes and lysosomes in melanoma A375 cells and HFF1 foreskin fibroblasts. Fig. S2. WX8 selectively disrupted macro‐autophagy in autophagy‐dependent cells. Fig. S3. Inhibitors of ER‐stress responses and their effect on melanoma A375 cell proliferation. Fig. S4. Induction of cell death by ectopic expression of IL24 was confirmed by accumulation of cells with less than normal amounts of DNA in G1 phase cells. Fig. S5. siRNA suppression of IL24 expression marginally reduced the sensitivity of melanoma A375 to WX8. Fig. S6. WX8‐induced noncanonical apoptosis in melanoma A375 cells. [file MOL2-18-988-s001.zip › Figure Captions.docx]

**Figure S1. Upregulation of genes linked to autophagosomes and lysosomes in melanoma A375 cells and HFF1 foreskin fibroblasts.** (**A**) Cells were cultured for 24 hours with vehicle, 0.05 μM WX8 (grey bars), or 1 μM WX8 (black bars). Total cell extracts were subjected to RNA sequence profiling (RNA-seq). Statistical significance was p < 0.05 (*), p < 0.001 (***), p < 0.0001 (****). Not significant was p > 0.05 (ns). (**B**) Upregulation of LC3-II, SQSTM1, LAMP1, CEBPb and JUN proteins was confirmed by immunoblotting. (**C**) Cells were cultured with WX8 for 4 hours, the time required to complete autophagy, in order to compare rates of autophagic flux [Roy et al., Autophagy 2023, PMID 36803256]. (**D**) LC3‑II/ACTB ratios were quantified from immunoblots in panel A.

**Figure S2. WX8 selectively disrupted macro-autophagy in autophagy-dependent cells.** (**A**) Upregulation of SQSTM1 and LAMP1 in cells cultured as in figure S1A was confirmed by RT-PCR. (**B**) PIKFYVE inhibitors disrupt lysosome homeostasis. This triggers an ER-stress response (black) in A375 cells that upregulates expression of glycosylated interleukin-24 (IL24). Excess IL24 amplifies the ER-stress response to induce cell death. Drugs such as thapsigargin and tunicamycin can also cause ER stress, but they are equally toxic to both normal and cancer cells. (**C**) Melanomas from 461 patient tumors (red) were significantly more enriched in IL24 RNA than 558 samples from normal skin (green). Average ratio of IL24 expressing tumors to normal skin was 9.1. Box is first to third quartile with a line through the median. Minimum and maximum values are indicated by lines. (Tang et al., Nucleic Acids Res. 2017, PMID 28407145).

**Figure S3. Inhibitors of ER-stress responses and their effect on melanoma A375 cell proliferation.** (**A**) Specific inhibitors of the three ER-stress pathways. (**B**) Sensitivity of A375 cell proliferation to inhibitors of ER-stress. (**C, E**) A375 cells were cultured for 24 hours with 5 μM thapsigargin (THAP) and the indicated concentrations of each of the EIF2AK3/PERK inhibitors in panel A. (**D**) A375 cells were cultured for 48 hours with 1 μM WX8 and the indicated concentrations of each EIF2AK3/PERK inhibitor in panel A.

**Figure S4**. **Induction of cell death by ectopic expression of IL24 was confirmed by accumulation of cells with less than normal amounts of DNA in G1 phase cells.** FACS analyses quantified the fraction of cells with <2N DNA content from melanoma A375 cells and Hs27 foreskin fibroblasts in figure 7B and 7E.

**Figure S5. siRNA suppression of IL24 expression marginally reduced the sensitivity of melanoma A375 to WX8.** (**A**) Melanoma A375 cells were cultured for 48 hours with either 10 nM nt-siRNA or siRNA against IL24 (siIL24) with either vehicle or 0.25 μM WX8. Whole cell lysate was used for immunoblotting for IL24 protein. (**B**) Photographs of the indicated cells in panel A prior to immunoblotting. (**C**) A375 cells treated with 10 nM nt-siRNA or siIL24 were cultured for 72 hours with the indicated concentrations of WX8. Cells were stained with trypan blue, and the fractions of live cells (unstained) and dead cells (stained) were quantified. Approximate IC50 values are indicated. (**D**) The fractions of dead cells in panel C are plotted as a function of their WX8 concentration. Standard deviations are indicated for duplicate experiments in panels C and D.

**Figure S6. WX8 induced non-canonical apoptosis in melanoma A375 cells. (A)** The fraction of A375 and HFF1 cells containing <2N DNA (less than G1 phase cells) after cultured them for 96 hours with the indicated concentrations of WX8. To allow a logarithmic axis, 0 μM WX8 (vehicle) was plotted as 0.001 μM or 0.1 μM WX8. (**B**) Cells were cultured with either 1 μM WX8 or Adriamycin (Adr) for 24 hours. Whole cell extracts were immuno-blotted to detect the indicated proteins based on their molecular weights and antibody affinity. (**C**) A375 cells were cultured with vehicle (V), WX8 or Adr for 24 hours. Samples were also treated with the general caspase inhibitor Z-VAD-fmk. (**D**) The fraction of live (trypan blue resistant) A375 cells in panel C was determined. Panel A was reproduced from Roy et al., Autophagy, 2023, PMID 36803256.
