# Supplementary figures and images for: PIKFYVE inhibitors trigger interleukin‐24‐dependent cell death of autophagy‐dependent melanoma
Source: Mol Oncol. 2024 Feb 27;18(4):988–1011. doi: 10.1002/1878-0261.13607 (PMC10994231; doi:10.1002/1878-0261.13607)

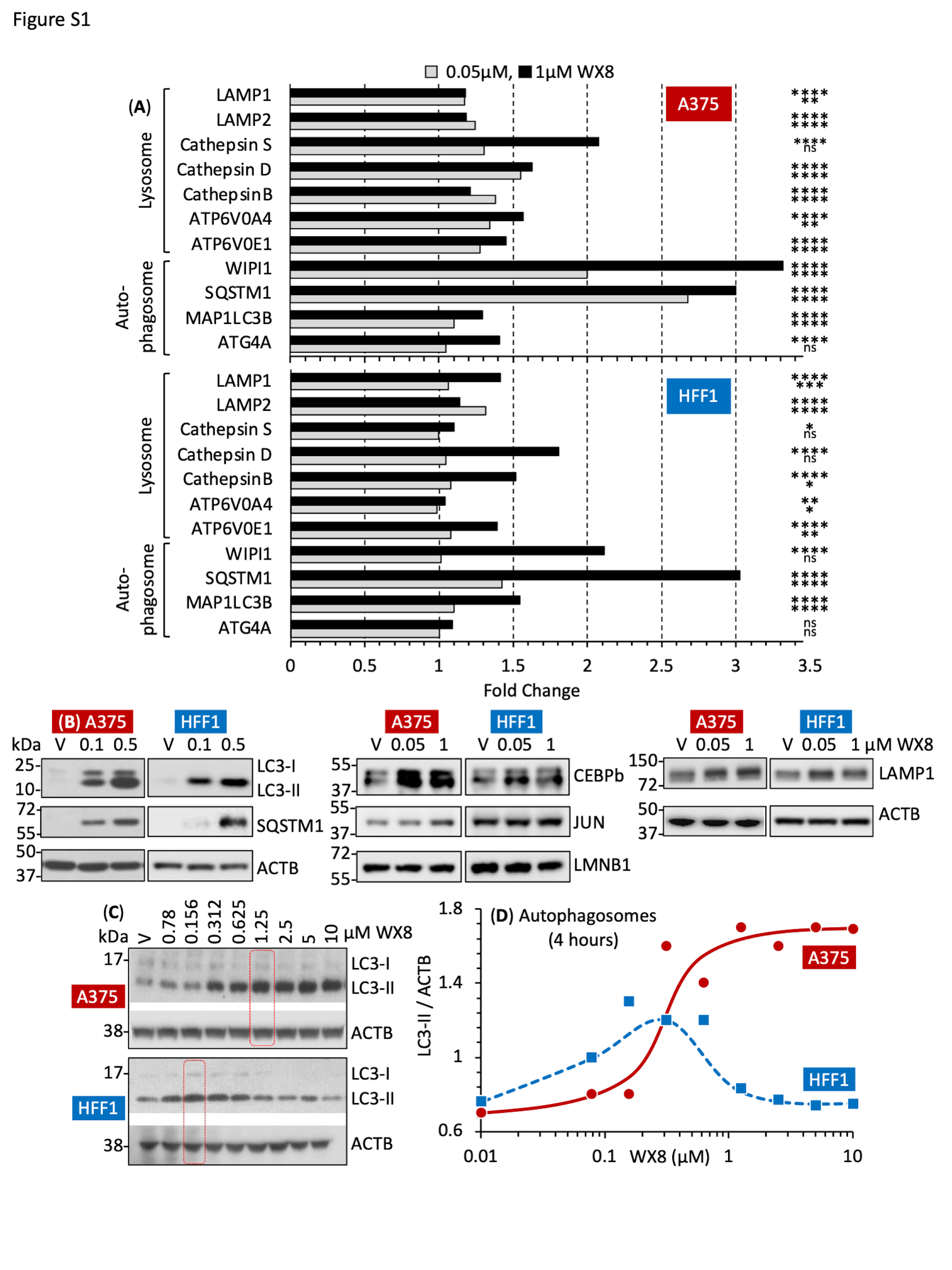

Supplement: Supplementary file 1 — Fig. S1. Upregulation of genes linked to autophagosomes and lysosomes in melanoma A375 cells and HFF1 foreskin fibroblasts. Fig. S2. WX8 selectively disrupted macro‐autophagy in autophagy‐dependent cells. Fig. S3. Inhibitors of ER‐stress responses and their effect on melanoma A375 cell proliferation. Fig. S4. Induction of cell death by ectopic expression of IL24 was confirmed by accumulation of cells with less than normal amounts of DNA in G1 phase cells. Fig. S5. siRNA suppression of IL24 expression marginally reduced the sensitivity of melanoma A375 to WX8. Fig. S6. WX8‐induced noncanonical apoptosis in melanoma A375 cells. [file MOL2-18-988-s001.zip › mol213607-sup-0001-FigureS1.tiff]

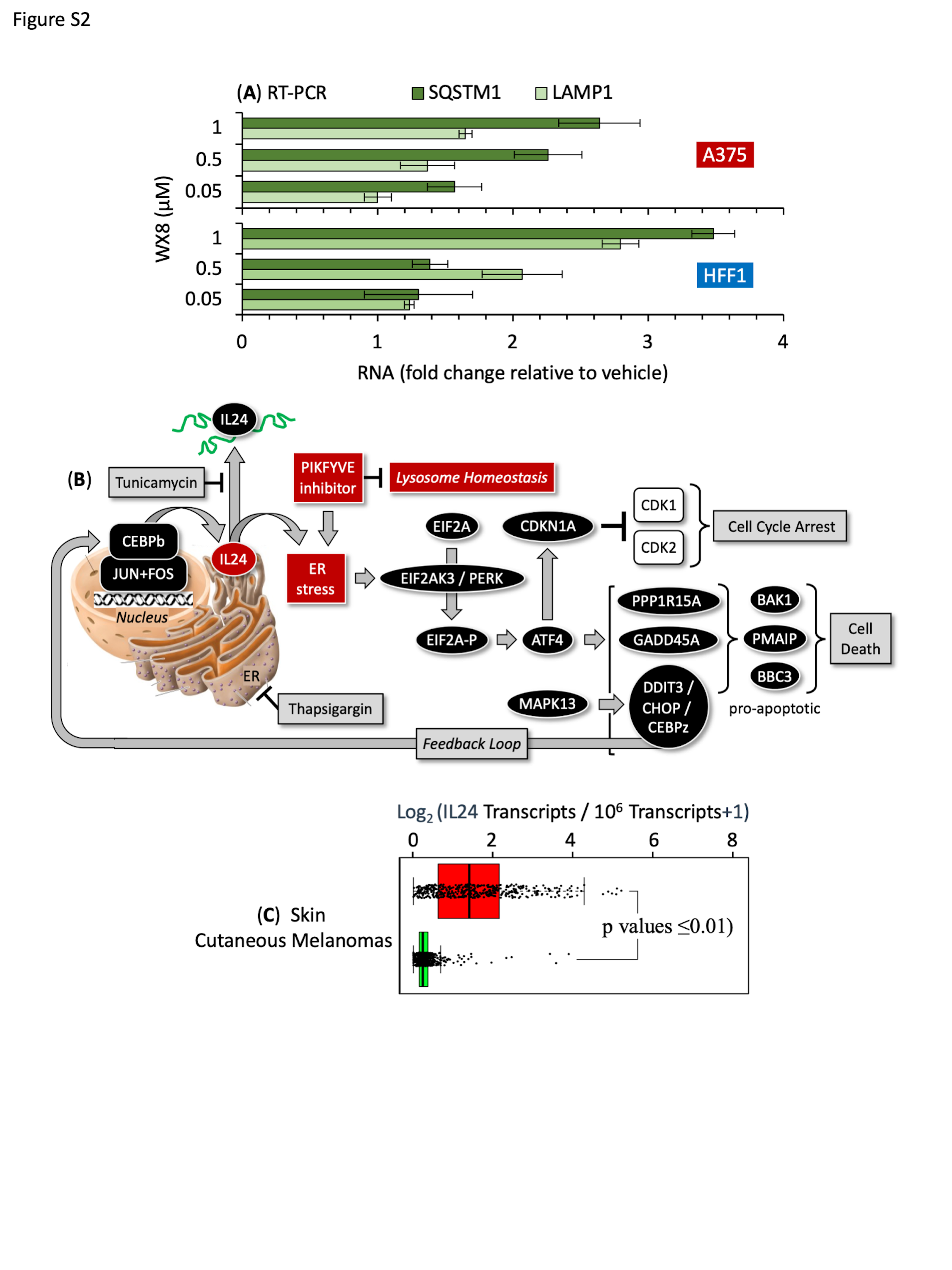

Supplement: Supplementary file 1 — Fig. S1. Upregulation of genes linked to autophagosomes and lysosomes in melanoma A375 cells and HFF1 foreskin fibroblasts. Fig. S2. WX8 selectively disrupted macro‐autophagy in autophagy‐dependent cells. Fig. S3. Inhibitors of ER‐stress responses and their effect on melanoma A375 cell proliferation. Fig. S4. Induction of cell death by ectopic expression of IL24 was confirmed by accumulation of cells with less than normal amounts of DNA in G1 phase cells. Fig. S5. siRNA suppression of IL24 expression marginally reduced the sensitivity of melanoma A375 to WX8. Fig. S6. WX8‐induced noncanonical apoptosis in melanoma A375 cells. [file MOL2-18-988-s001.zip › mol213607-sup-0002-FigureS2.tiff]

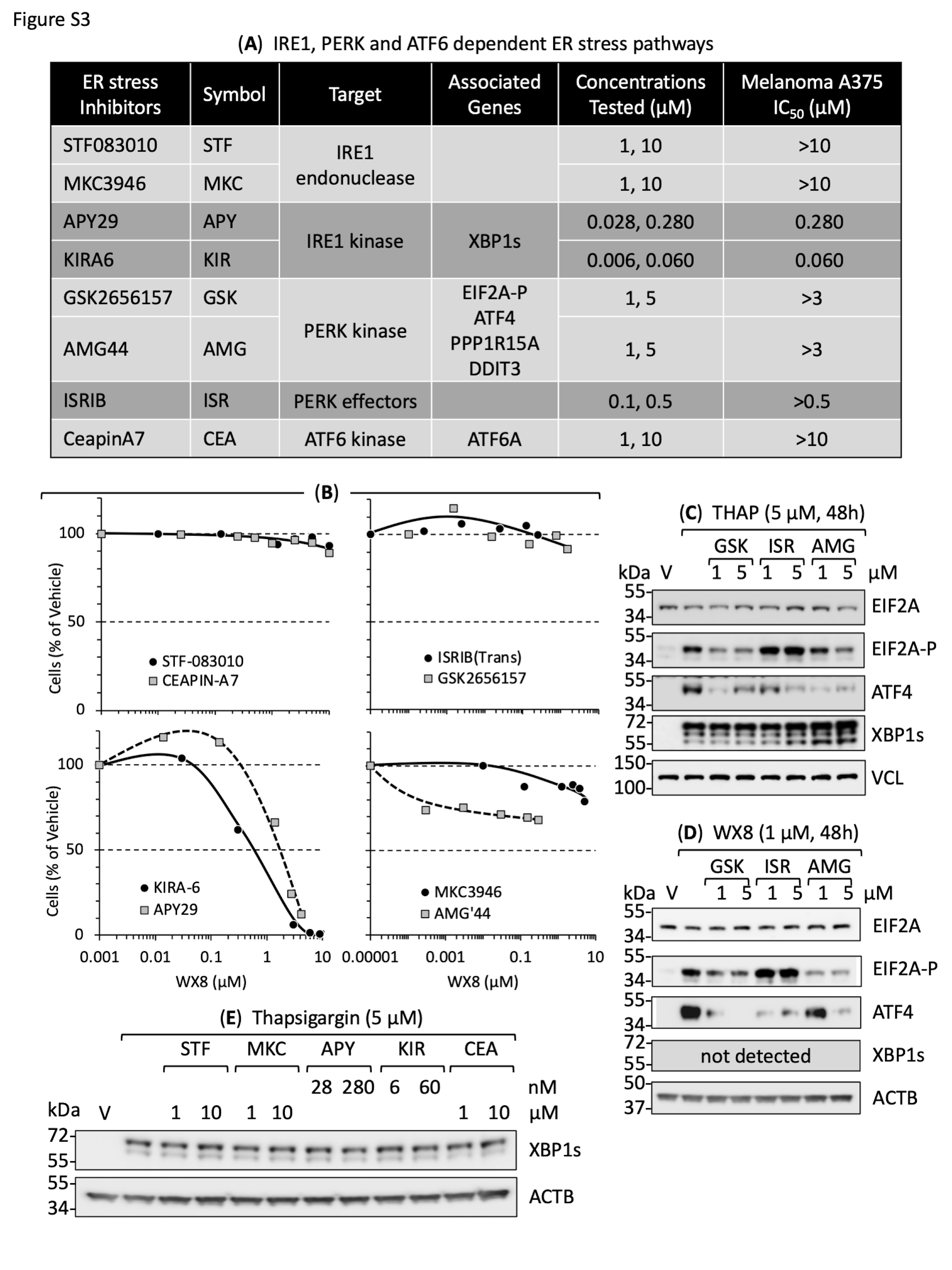

Supplement: Supplementary file 1 — Fig. S1. Upregulation of genes linked to autophagosomes and lysosomes in melanoma A375 cells and HFF1 foreskin fibroblasts. Fig. S2. WX8 selectively disrupted macro‐autophagy in autophagy‐dependent cells. Fig. S3. Inhibitors of ER‐stress responses and their effect on melanoma A375 cell proliferation. Fig. S4. Induction of cell death by ectopic expression of IL24 was confirmed by accumulation of cells with less than normal amounts of DNA in G1 phase cells. Fig. S5. siRNA suppression of IL24 expression marginally reduced the sensitivity of melanoma A375 to WX8. Fig. S6. WX8‐induced noncanonical apoptosis in melanoma A375 cells. [file MOL2-18-988-s001.zip › mol213607-sup-0003-FigureS3.tiff]

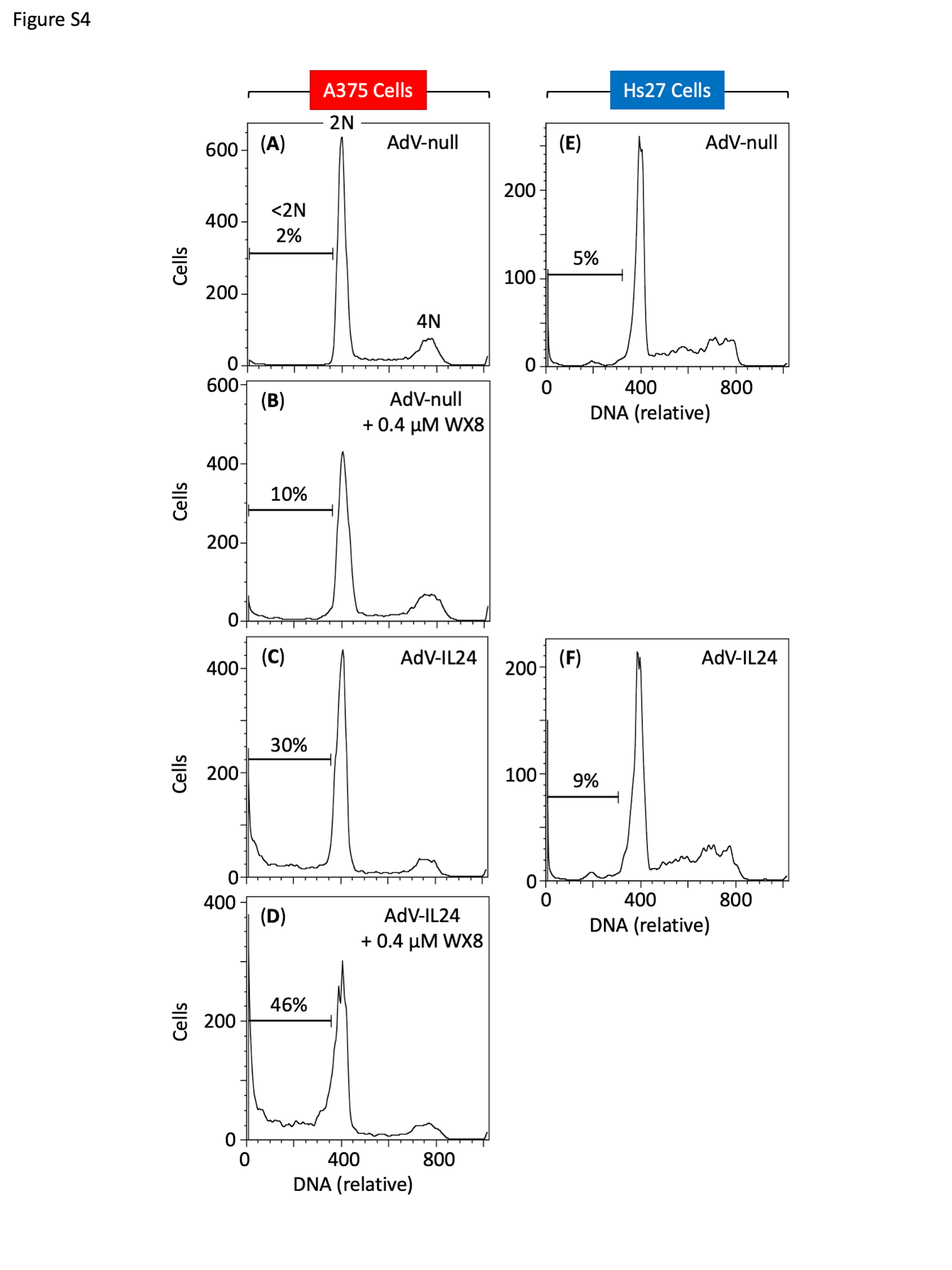

Supplement: Supplementary file 1 — Fig. S1. Upregulation of genes linked to autophagosomes and lysosomes in melanoma A375 cells and HFF1 foreskin fibroblasts. Fig. S2. WX8 selectively disrupted macro‐autophagy in autophagy‐dependent cells. Fig. S3. Inhibitors of ER‐stress responses and their effect on melanoma A375 cell proliferation. Fig. S4. Induction of cell death by ectopic expression of IL24 was confirmed by accumulation of cells with less than normal amounts of DNA in G1 phase cells. Fig. S5. siRNA suppression of IL24 expression marginally reduced the sensitivity of melanoma A375 to WX8. Fig. S6. WX8‐induced noncanonical apoptosis in melanoma A375 cells. [file MOL2-18-988-s001.zip › mol213607-sup-0004-FigureS4.tiff]

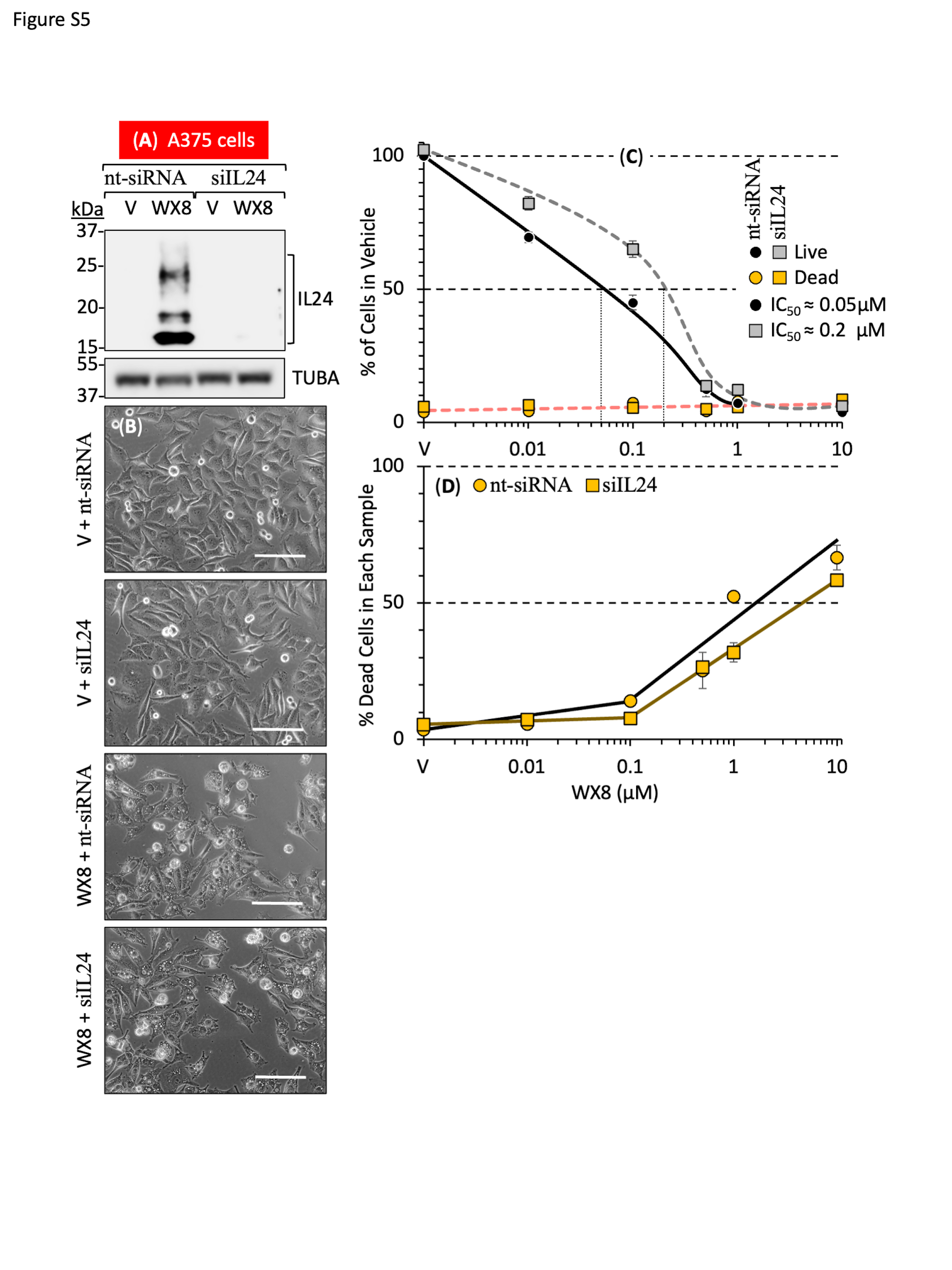

Supplement: Supplementary file 1 — Fig. S1. Upregulation of genes linked to autophagosomes and lysosomes in melanoma A375 cells and HFF1 foreskin fibroblasts. Fig. S2. WX8 selectively disrupted macro‐autophagy in autophagy‐dependent cells. Fig. S3. Inhibitors of ER‐stress responses and their effect on melanoma A375 cell proliferation. Fig. S4. Induction of cell death by ectopic expression of IL24 was confirmed by accumulation of cells with less than normal amounts of DNA in G1 phase cells. Fig. S5. siRNA suppression of IL24 expression marginally reduced the sensitivity of melanoma A375 to WX8. Fig. S6. WX8‐induced noncanonical apoptosis in melanoma A375 cells. [file MOL2-18-988-s001.zip › mol213607-sup-0005-FigureS5.tiff]

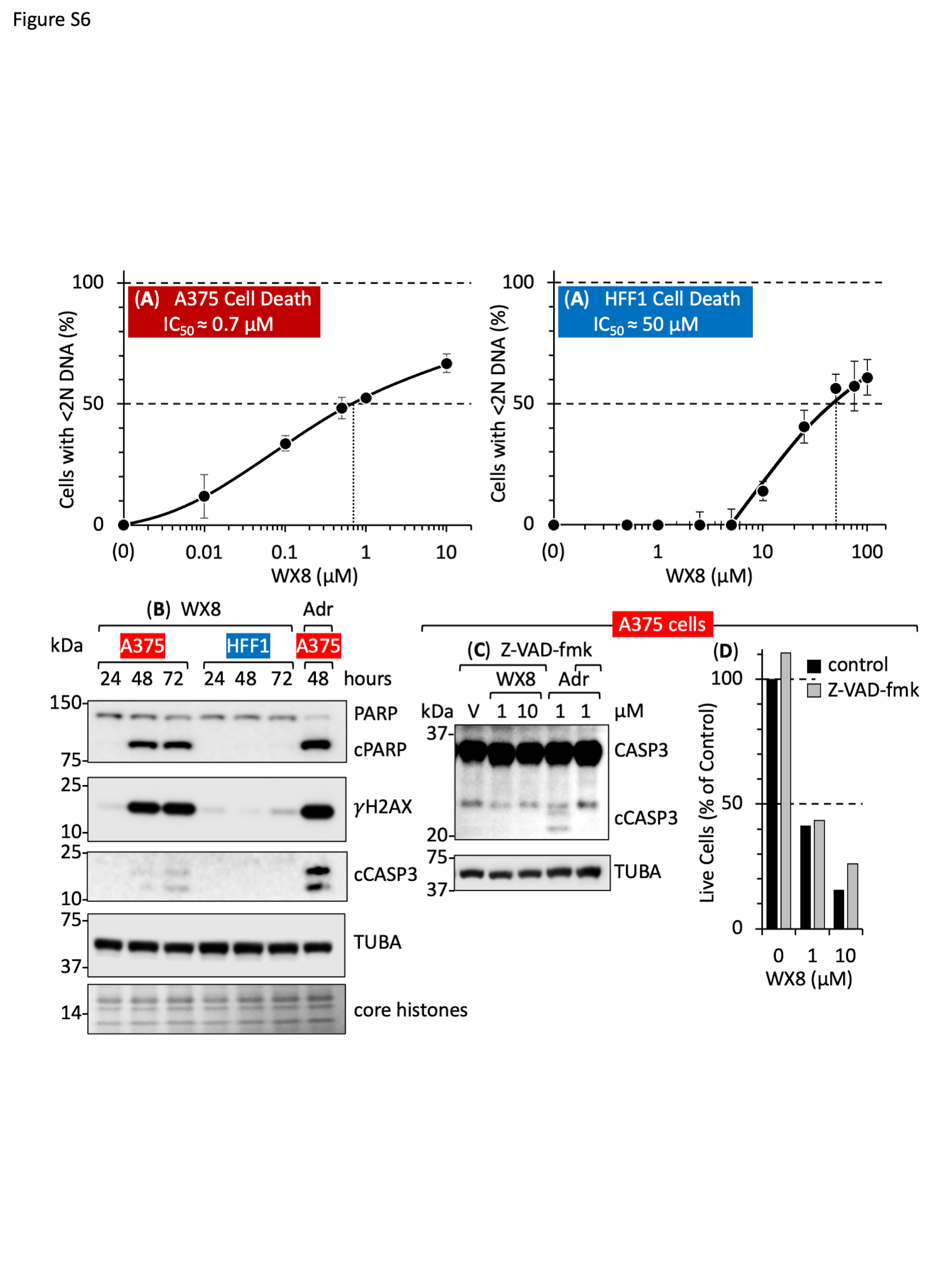

Supplement: Supplementary file 1 — Fig. S1. Upregulation of genes linked to autophagosomes and lysosomes in melanoma A375 cells and HFF1 foreskin fibroblasts. Fig. S2. WX8 selectively disrupted macro‐autophagy in autophagy‐dependent cells. Fig. S3. Inhibitors of ER‐stress responses and their effect on melanoma A375 cell proliferation. Fig. S4. Induction of cell death by ectopic expression of IL24 was confirmed by accumulation of cells with less than normal amounts of DNA in G1 phase cells. Fig. S5. siRNA suppression of IL24 expression marginally reduced the sensitivity of melanoma A375 to WX8. Fig. S6. WX8‐induced noncanonical apoptosis in melanoma A375 cells. [file MOL2-18-988-s001.zip › mol213607-sup-0006-FigureS6.tiff]
